# Two Novel *S*‐methyltransferases Confer Dimethylsulfide Production in *Actinomycetota*

**DOI:** 10.1002/advs.202510141

**Published:** 2025-12-03

**Authors:** Ruihong Guo, Zihua Guo, Yi Zhou, Yunhui Zhang, Haojin Cheng, Rebecca Devine, Chuang Sun, Ronghua Liu, Yanfen Zheng, Andrew J. Gates, Jonathan D. Todd, Xiao‐Hua Zhang

**Affiliations:** ^1^ Frontiers Science Center for Deep Ocean Multispheres and Earth System, and College of Marine Life Sciences Ocean University of China Qingdao 266003 China; ^2^ Laboratory for Marine Ecology and Environmental Science Qingdao Marine Science and Technology Center Qingdao 266003 China; ^3^ Key Laboratory of Evolution and Marine Biodiversity (Ministry of Education), and Institute of Evolution and Marine Biodiversity Ocean University of China Qingdao 266003 China; ^4^ John Innes Centre Department of Molecular Microbiology, and Centre for Microbial Interactions Norwich Research Park Norwich NR4 7UH UK; ^5^ School of Biological Sciences University of East Anglia Norwich Research Park Norwich NR4 7TJ UK; ^6^ Marine Agriculture Research Center Tobacco Research Institute of Chinese Academy of Agricultural Sciences Qingdao 266101 China

**Keywords:** *Actinomycetota*, dimethylsulfide, methanethiol, *S*‐methyltransferases, sulfide

## Abstract

Hydrogen sulfide (H_2_S), methanethiol (MeSH), and dimethylsulfide (DMS) are abundant sulfur gases with crucial roles in global sulfur cycling, chemotaxis, and climate regulation. Microorganisms can *S*‐methylate H_2_S and MeSH, which can be cytotoxic, to yield non‐toxic DMS via MddA or MddH enzymes in largely terrestrial or marine environments, respectively. However, the potential of many important and abundant bacteria like *Actinomycetota* is underestimated due to unknown Mdd enzymes. Here, two novel *S*‐adenosine‐methionine‐dependent H_2_S and MeSH *S*‐methyltransferases, MddM1 and MddM2 are identified, in the DMS‐producing actinomycete *Mycolicibacterium poriferae* (*M. poriferae*) ZYF656, isolated from the Mariana Trench. *M. poriferae* ZYF656 MddM1 and MddM2 likely detoxify H_2_S and MeSH and alleviate oxidative stress, since *mddM1* and *mddM2* transcription is induced by H_2_S, MeSH, and oxidative stress, and their expression in *E. coli* enhances H_2_S, MeSH, and oxidative stress tolerance. MddM1 and/or MddM2 are in >50% of *actinomycetota*, including the model *Streptomyces* species, *S. venezuelae*, but are also seen in some *Chloroflexota*, *Acidobacteriota*, and *Proteobacteria*. *mddM1* is always more abundant than *mddM2* in diverse environments and is prevalent in soils and marsh sediments. This study highlights the significance of H_2_S‐ and MeSH‐dependent DMS production and, principally, of *Actinomycetota* in global DMS production and sulfur cycling.

## Graphical abstract

This study identifies two novel *S*‐adenosine‐methionine‐dependent methyltransferases, MddM1 and MddM2, in actinomycetes from the Mariana Trench. These enzymes can convert toxic hydrogen sulfide (H_2_S) and methanethiol (MeSH) into dimethylsulfide (DMS), serving as a cellular detoxification and oxidative stress response. *mddM1* gene is widespread in *Actinomycetota* and diverse environments, revealing their underestimated, crucial roles in global sulfur cycling and DMS production.

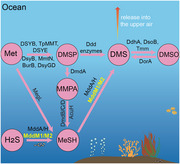

## 1 Introduction

Dimethylsulfide (DMS) is Earth's major biogenic sulfur compound transferred from marine environments to the atmosphere, representing 13–37 Tg sulfur annually.^[^ ^1^, ^2^, ^3^, ^4^ ^]^ Atmospheric DMS and related compounds, like methanethiol (MeSH), can be oxidized to form cloud condensation nuclei and impact the climate.^[^ ^5^, ^6^, ^7^ ^]^ Furthermore, DMS is a nutrient and a signaling molecule for diverse microorganisms.^[^ ^8^, ^9^ ^]^

**Figure 1:**
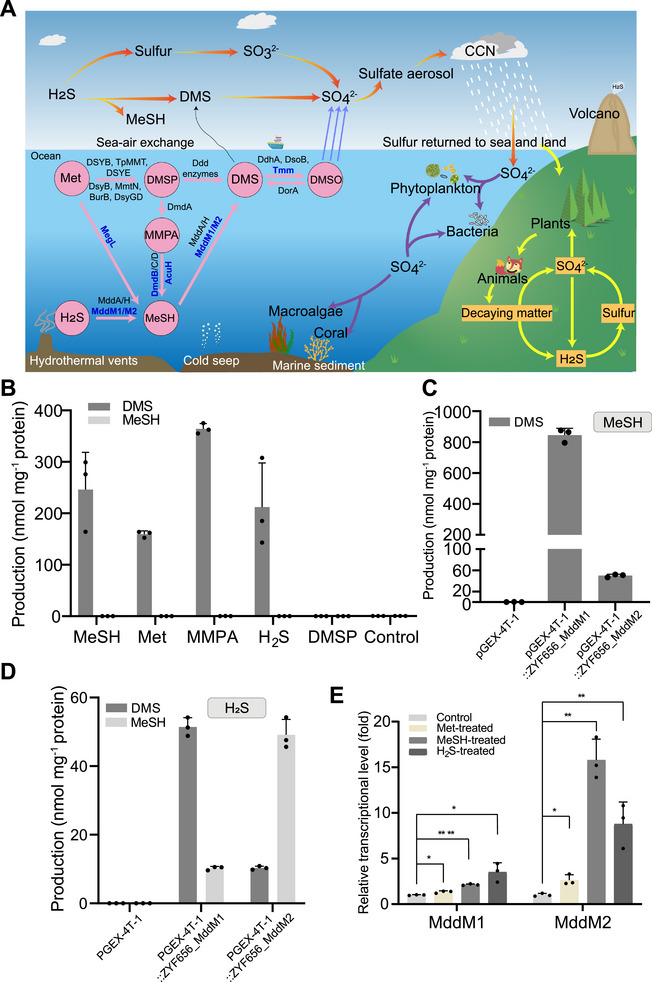
Analysis of H_2_S and MeSH *S*‐methylation by *M*. *poriferae* ZYF656 and its candidate Mdd enzymes. A) Simplified DMSP/DMS cycle and the key enzymes/pathways involved. Blue fonts predict enzymes/pathways in the strain *M*. *poriferae* ZYF656. B) Gas chromatography detection of DMS and MeSH produced from *M*. *poriferae* ZYF656 when incubated with 0.5 mm Met, MeSH, MMPA, H_2_S, DMSP, or a negative control. C) DMS production from *E. coli* BL21(DE3) with an empty vector or with clones expressing cloned *mddM1*, *mddM2*, when grown with 0.5 mm MeSH in M9 media. D) MeSH and DMS production from *E. coli* BL21(DE3) containing cloned *mddM1*, *mddM2*, or empty vector, when grown with 0.5 mm H_2_S in M9 media. E) RT‐qPCR analyzes of *mddM1* and *mddM2* in *M*. *poriferae* ZYF656 grown with 0.5 mm Met, MeSH, or H_2_S. The values for DMS and MeSH production are shown as mean ± s.d., and with three biological replicates for each strain. Significance was determined by Student's *t*‐test (*p < 0.05, **p < 0.01, ***p < 0.001, ****p < 0.0001).

In marine environments, most DMS is thought to arise from the microbial cleavage of the abundant osmolyte dimethylsulfoniopropionate (DMSP) via diverse DMSP lyase enzymes.^[^ ^10^ ^]^ However, DMS can also result from the enzymatic degradation of dimethyl sulfoxide (DMSO), methoxyaromatic compounds,^[^ ^11^ ^]^ or from *S*‐methylation of MeSH or hydrogen sulfide (H_2_S).^[^ ^12^, ^13^, ^14^, ^15^ ^]^ H_2_S is a toxic volatile, often present in diverse environments, for example, hydrothermal vents and sediments, at millimolar concentrations.^[^ ^16^, ^17^ ^]^ MeSH, another toxic volatile, is a potentially abundant by‐product of DMSP demethylation, which accounts for up to 80% of marine DMSP catabolism (**Figure** **1**A).^[^ ^18^ ^]^ In this pathway, DMSP is demethylated to 3‐methylmercaptopropionate (MMPA) via DmdA, predicted in 20% of marine bacteria.^[^ ^19^, ^20^ ^]^ MMPA can be further processed to MeSH via *dmdB/C/D* gene products which are ubiquitous in marine and terrestrial bacteria (Figure 1A).^[^ ^19^, ^20^ ^]^ MeSH can also result from DMS and methionine (Met) degradation via DMS monooxygenase DmoA and Met‐gamma‐lyase enzyme MegL, respectively (Figure 1A).^[^ ^21^, ^22^, ^23^ ^]^ Thus, there are many DMSP‐independent routes to DMS, and the substrates for these pathways, particularly H_2_S and MeSH, are often abundant in marine and terrestrial environments.

Previous studies identified TMT1A and TMT1B as thiol methyltransferase enzymes capable of methylating H_2_S in humans, other mammals, and fish.^[^ ^24^, ^25^ ^]^ Furthermore, diverse microorganisms utilized enzymes, termed MddA and MddH, to *S*‐methylate H_2_S and MeSH yielding DMS in reactions where *S*‐adenosine methionine (SAM) was the methyl donor (Figure 1A).^[^ ^14^, ^15^ ^]^ These Mdd enzymes were proposed to detoxify H_2_S and MeSH via the production of non‐toxic DMS.^[^ ^14^, ^15^ ^]^ MddA, a membrane‐associated enzyme, was predominantly found in terrestrial actinobacteria, cyanobacteria, rhizobiales, pseudomonads, and some marine algae.^[^ ^26^ ^]^ In contrast, MddH was shown to be a cytoplasmic enzyme and widespread in diverse marine bacteria, especially *Gammaproteobacteria*.^[^ ^15^ ^]^ The genetic potential to *S*‐methylate H_2_S and MeSH was found to be sizable in diverse marine (largely via MddH) and terrestrial (largely via MddA) environments, particularly in sediments, where H_2_S and MeSH concentrations were likely higher.^[^ ^16^, ^17^ ^]^ Although *mddH* was abundant in marine multi‐omics data, it was never more abundant than DMSP lyase genes.^[^ ^15^ ^]^ These findings implied that microbial *S*‐methylation of H_2_S and MeSH played important roles in microbial stress responses and global DMS production, but less so than DMSP cleavage in marine settings. However, there were still bacteria that *S*‐methylated H_2_S and MeSH but lacked MddA and MddH and thus contained novel enzymes.^[^ ^12^, ^13^, ^15^ ^]^

Here, we screened cultivable bacteria from the Mariana Trench, previously reported to exhibit high Mdd activity,^[^ ^27^ ^]^ for isolates that *S*‐methylated H_2_S and MeSH. One such isolate, the actinomycete *Mycolicibacterium poriferae* (*M. poriferae*) ZYF656, was able to *S*‐methylate H_2_S and MeSH but lacked all known *mdd* genes, *TMT1A* and *TMT1B*, implying it contained novel Mdd enzymes. We identified and characterized two novel enzymes responsible for H_2_S and MeSH *S*‐methylation, their roles in bacteria, and implied importance in stress tolerance, global DMS production, and sulfur cycling.

## 2 Results

### 2.1 DMS and MeSH Production by *Mycolicibacterium poriferae* ZYF656

We noted that *M. poriferae* ZYF656, a new species isolated from the 9600 m deep Mariana Trench seawater, produced DMS when incubated with Met, MMPA, H_2_S, and MeSH (Figure 1B). This actinobacterium did not produce DMSP even when grown in the presence of Met (the universal DMSP precursor), and it failed to produce DMS or MeSH when incubated with DMSP (Figure 1B). These data confirmed that *S*‐methylation of H_2_S and MeSH, rather than DMSP‐dependent pathways was the source of DMS produced by this marine actinobacterium.

The *M. poriferae* ZYF656 genome (GenBank accession number: CP151154) lacked both identifiable DMSP demethylase (*dmdA*) and DMSP lyase (*ddd*) genes (Figure 1A), consistent with its absence of DMSP lyase and demethylation activity. However, *M. poriferae* ZYF656 did contain *megL* and *dmdB/AcuH*, whose Met‐gamma‐lyase (EC4.4.1.11) and DMSP demethylation pathway protein products can generate MeSH from Met and MMPA, respectively (Figure 1A, Table S1, Supporting Information). These findings are consistent with the data shown in Figure 1B, supporting the proposed role of Met and MMPA as precursors for DMS production via MeSH *S*‐methylation. Interestingly, the predicted *M. poriferae* ZYF656 proteome lacked any obvious MddA, MddH, TMT1A, or TMT1B homologues at ≥40% amino acid identity, implying that this actinobacterium may utilize novel Mdd enzymes for H_2_S and MeSH *S*‐methylation.

### 2.2 Identification of the Novel Enzymes MddM1 and MddM2

To identify the genes responsible for H_2_S and MeSH *S*‐methylation, a genomic library of *M. poriferae* ZYF656 was constructed and screened in *Escherichia coli* (*E. coli*) JM109 for Mdd activity. Two distinct clones with Mdd activity were identified and sequenced. Each clone contained a distinct SAM‐dependent methyltransferase, termed MddM1 (PP661493) and MddM2 (PP661494). When cloned and expressed in *E. coli* BL21 (DE3), *mddM1* and *mddM2* each conferred the ability to *S*‐methylate H_2_S to MeSH and DMS, as well as MeSH to DMS (Figure 1C,D). Furthermore, cloned *mddM1* and *mddM2* each also conferred MeSH‐dependent DMS production from 1 mm MeSH and H_2_S to *Corynebacterium glutamicum* (*C. glutamicum*) RES167, an actinobacterium lacking the Mdd pathway (Figure S1A,B, Supporting Information).

MddM1 and MddM2 were similar‐sized proteins comprising 210 and 204 amino acids with predicted molecular weights of ≈23 and ≈21 kDa, respectively. However, their theoretical *p*I values were quite different being 5.88 for MddM1 and 7.86 for MddM2. MddM1 was a UbiG family (COG2227) SAM‐dependent methyltransferase, predicted by CELLO to be cytoplasmic with no signal peptide. Like MddH,^[^ ^15^ ^]^ MddM2 was a UbiE family SAM‐dependent methyltransferase, but by contrast, it was predicted to be membrane‐bound (by CELLO), like MddA,^[^ ^14^ ^]^ with a transmembrane helix (by TMHMM) and a SEC signal peptide whose cleavage site was likely between position residues 41 and 42 as determined by SignalP 6.0.^[^ ^28^ ^]^ Indeed, *M. poriferae* ZYF656 MeSH *S*‐methylation activity was found to be both cytosolic (1.34 ± 0.32 pmol DMS mg^−1^ total protein h^−1^) and membranous (0.17 ± 0.01 pmol DMS mg^−1^ total protein h^−1^), consistent with the higher activity of the MddM1 protein and lower activity of the MddM2 protein.

Sequence alignment of MddM1 and MddM2 with other characterized SAM‐dependent *S*‐methyltransferases, including microbial MddH and human TMT1A and TMT1B, revealed that they shared extended residue similarity including the conserved GxGxG motif for SAM binding (Figure S2A, Supporting Information).^[^ ^15^ ^]^ Further structural modelling, using AlphaFold3,^[^ ^29^ ^]^ showed MddM1 and MddM2 also shared structural similarity with each other and with other characterized SAM‐dependent methyltransferases, but they have different N‐terminal regions (Figure S2B, Supporting Information). While the N‐terminal region of MddM1 is predicted to adopt a similar structure to MddH that is truncated relative to human SAM‐dependent *S*‐methyltransferases,^[^ ^15^ ^]^ the N‐terminal region of MddM2 is predicted to be an extended unstructured peptide region containing the putative SEC signal sequence. As MddM1 and MddM2 adopt an MddH‐like fold, both are structurally distinct to the microbial integral membrane SAM‐dependent *S*‐methyltransferase MddA.^[^ ^26^ ^]^

### 2.3 MddM1 and MddM2 are Abundant in *Actinomycetota*

**Figure 2:**
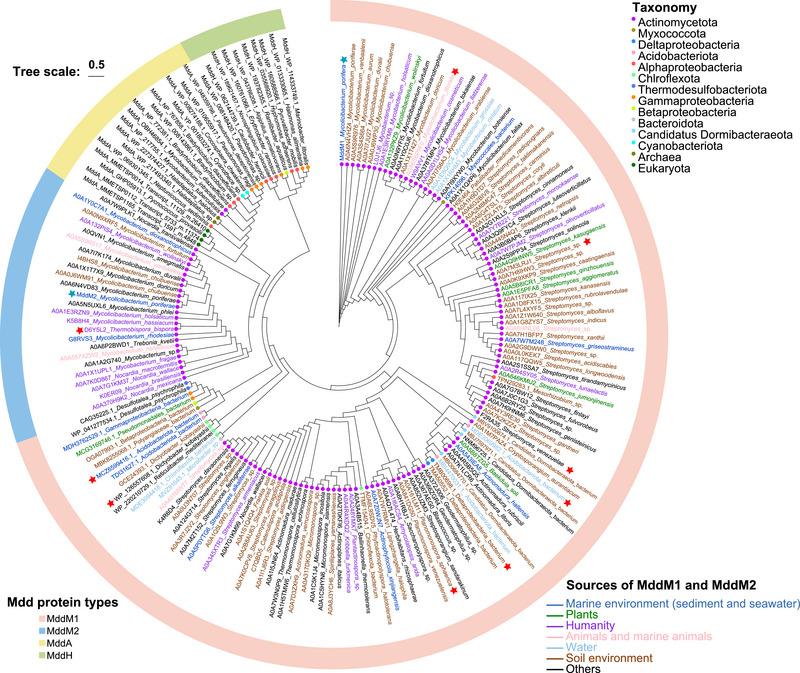
Maximum‐likelihood phylogenetic tree of MddM proteins. The tree was constructed using IQ‐Tree using the general time reversible model with empirical frequencies and three rates (LG + F + G4), together with the proteins previously shown to have the expected *S*‐methyltransferase enzyme activity for DMS production. The scale bar indicates 0.5 amino acid substitutions per site. MddM1 and MddM2 from *M. poriferae* ZYF656 are highlighted by a blue star. Methyltransferase enzymes with experimentally determined Mdd activity are highlighted with a red star. The evolutionary tree uses three distinct color schemes to represent different types of information: The color blocks around the individual proteins indicate the different Mdd proteins (See Mdd protein types Key). The round dots on the branches indicate the taxonomic classification of the bacterial strains (see Taxonomy Key). The color of the leaf labels (organism names) indicates the source of the sequences (see Source Key).

The distribution of *mddM1* and *mddM2* in genomes available on the UniprotKB and Swiss‐Prot database was analyzed to predict organisms with the potential to *S*‐methylate H_2_S and MeSH. Candidate MddM1 and MddM2 proteins (*E*‐value ≤ e‐30) were predominantly found in *Actinomycetota*, but also in some *Proteobacteria* (including *Alpha*‐, *Beta*‐, *Gamma*‐ and *Delta‐proteobacteria*), *Chloroflexota*, *Myxococcota*, *Acidobacteriota*, *Desulfuromonadia*, and *Candidatus* Dormibacteraeota (**Figure** **2**). These MddM1 homologues were predominantly from soil or marine environments, but were also seen, albeit less frequently, in bacteria from human, plant, animal, water, and other sources. In contrast, only 24 MddM2 homologues were identified, and all were *Actinomycetota* from human sources (Figure 2). Representative candidate MddM1 and MddM2 homologues from *Actinomycetota*, *Acidobacteriota*, *Deltaproteobacteria*, and *Chloroflexota* were overexpressed in *E. coli* BL21 (DE3), and all showed H_2_S‐ and MeSH‐dependent DMS production, confirming the activity of these proteins (**Table** **1**).

**Table 1:** Activity of diverse MddM proteins expressed in E. coli BL21(DE3). Diverse MddM proteins were cloned into the pET‐24a vector and expressed in *E. coli* BL21 (DE3) grown with 0.5 mm MeSH or with 0.5 mm H_2_S. Sequences not highlighted in grey are MddM1 homologues. MddM2 homologue is highlighted in grey.

Accession number	Source organism	MeSH	H_2_S	
		nmol DMS h^−1^ mg total protein^−1^	nmol MeSH h^−1^ mg total protein^−1^	nmol DMS h^−1^ mg total protein^−1^
MCZ6599416.1	*Acidobacteriota bacterium*	33.23 ± 0.46	33.16 ± 0.82	10.65 ± 0.29
MDE3069982.1	*Acidobacteriota bacterium*	597.19 ± 6.01	27.22 ± 3.73	9.18 ± 0.79
A0A6S6P9F0	*Mycolicibacterium litorale*	107.41 ± 22.41	43.02 ± 3.52	14.57 ± 0.35
TMB00698.1	*Deltaproteobacteria bacterium*	14.29 ± 1.82	112.66 ± 11.25	84.72 ± 9.03
WP_126557608.1	*Dictyobacter kobayashii*	580.93 ± 2.43	45.60 ± 0.52	14.91 ± 1.02
A0A7M3LRJ1	*Streptomyces* sp. SAJ15	26.86 ± 2.88	0	8.79 ± 0.97
A0A161LM11	*Planomonospora sphaerica*	48.97 ± 4.54	30.38 ± 2.89	7.92 ± 0.35
F2RA35	*Streptomyces venezuelae*	76.03 ± 4.16	7.84 ± 0.86	4.89 ± 0.17
D6Y5L2	*Thermobispora bispora*	26.28 ± 0.43	51.77 ± 5.36	7.37 ± 0.16

The values for DMS or MeSH production are shown as mean ± s.d. for three biological replicates.

Focused analysis of 42815 *Actinomycetota* genomes available on NCBI inferred H_2_S and MeSH‐dependent DMS production to be important in this phylum, with 51.02%, 21.60%, 4.58%, and 1.16% predicted to contain MddM1, MddA, MddH, or MddM2, respectively (Figure S3, Supporting Information). The majority of MddM1 homologues were from *Streptomyces*, *Mycobacterium*, and *Mycolicibacterium* genera (Figure S3, Supporting Information). Notably, *Streptomyces* are generally reported as fast‐growing bacteria, widely distributed in diverse environments, particularly soils, and 19.75% contained MddM1 (Figure S3, Supporting Information).^[^ ^30^, ^31^ ^]^ These data implied that the *Actinomycetota*, particularly *Streptomyces* in soil and human environments may be significant producers of DMS via the *S*‐methylation of H_2_S and MeSH (Figure S3, Supporting Information). However, we found that the model *Streptomyces*, *S. venezuelae*, and its *mddM1* mutant derivative (generated here), made no DMS, even when incubated with Met or MeSH (Figure S1C,D, Supporting Information). However, DMS production was detected when *mddM1* was expressed under control of an ectopic promoter in the *S. venezuelae mddM1^−^* grown in the presence of Met or MeSH (Figure S1C,D, Supporting Information). These data implied that *S. venezuelae* did not express and utilize MddM1 under the tested conditions, as is common in *Streptomyces* secondary metabolite production systems.^[^ ^32^ ^]^ It also further indicated the limitation of functional predictions based only on genetic potential.

Since some eukaryotic algae contain MddA,^[^ ^14^ ^]^ we probed the Marine Microbial Eukaryote Transcriptome Sequencing Project (MMETSP) database with MddM1, MddM2, and MddH sequences.^[^ ^33^ ^]^ This identified 14 proteins clustered in a distinct clade away from MddM1 and MddH, and no MddM2 homologs (Figure S4, Supporting Information). To establish if the distinct clade of Mdd‐like proteins had H_2_S or MeSH *S*‐methylation activity, a representative from a liverwort *Riccia sorocarpa* (MMETSP0818 10907|m. 27989) with 41.30% amino acid identity, 95% coverage, and an *E*‐value of 2.68e‐34 to MddM1 (MDE3069982.1 *Acidobacteriota* bacterium), was chosen for characterization. This candidate gene was codon‐optimized, cloned into pET‐24a, and expressed in *E. coli* BL21 (DE3), but despite a soluble protein product being overproduced, it conferred no H_2_S or MeSH *S*‐methyltransferase activity. The absence of *S*‐methyltransferase activity in *E. coli* expressing the liverwort Mdd‐like gene may have been due to the protein product not folding correctly and/or a lack of appropriate co‐factors for activity in this heterologous host. Alternatively, these algal proteins may not constitute genuine Mdd enzymes, despite them containing the same methyltransferase family Pfam domain seen in MddH and MddM1. The function of these Mdd‐like proteins remains unknown, requires further investigation, and again highlights the importance of substantiating genomic predictions with functional analysis.

### 2.4 Characterization of Recombinant MddM1 and MddM2

**Figure 3:**
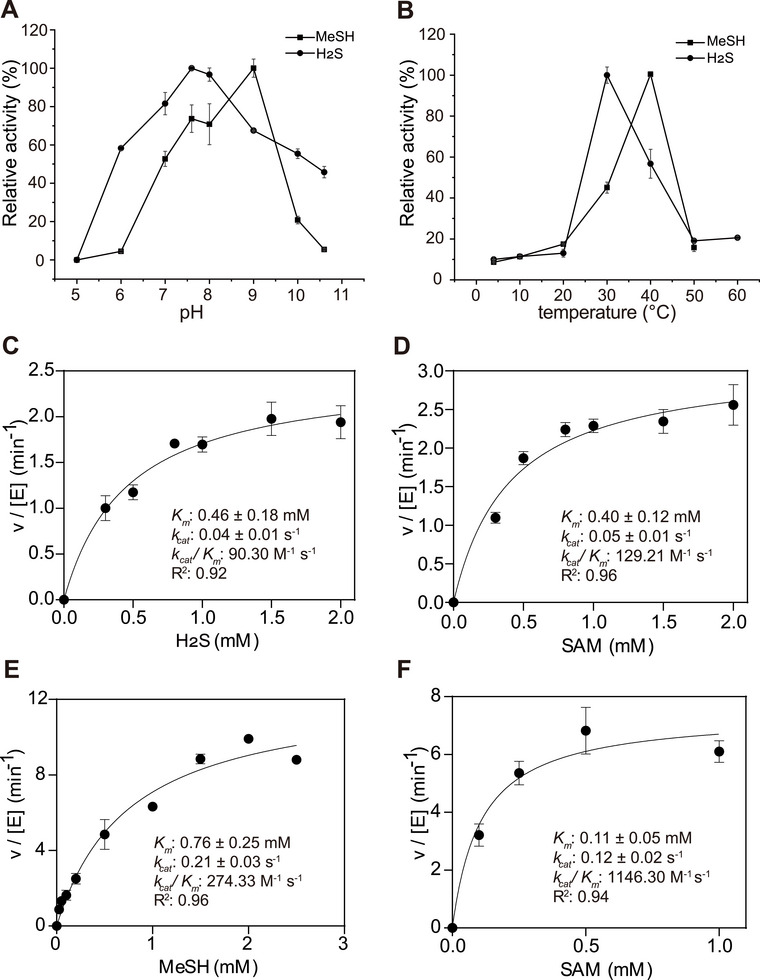
Kinetic characterization of recombinant MddM1. Effect of pH (A) and temperature (B) on the enzymatic activity of MddM1. The 100% activity values correspond to 251.19 and 286.16 nmol mg protein^−1^ min^−1^ for MeSH and H_2_S, respectively, at optimum pH, and to 209.12 and 194.66 nmol mg protein^−1^ min^−1^ at optimum temperature. Substrate‐dependence of MddM1 catalytic activity with varying H_2_S concentration (C), or SAM (D) when using H_2_S as a co‐substrate. Assays with H_2_S used 1 µg MddM1 at pH 7.5 and 30 °C. Substrate‐dependence of MddM1 activity with varying MeSH concentration (E), or SAM (F) when using MeSH as a co‐substrate. Assays with MeSH used 1 µg MddM1 at pH 9 and 40 °C. Kinetic constants reported in the data panels were obtained by non‐linear fitting of data using the rectangular‐hyperbola form of the Michaelis–Menten equation, where *v*/[E] = *k*_cat_ × [S]/(*K*_m_ + [S]). The values for DMS production are shown as mean ± s.d. for three biological replicates.

**Figure 4:**
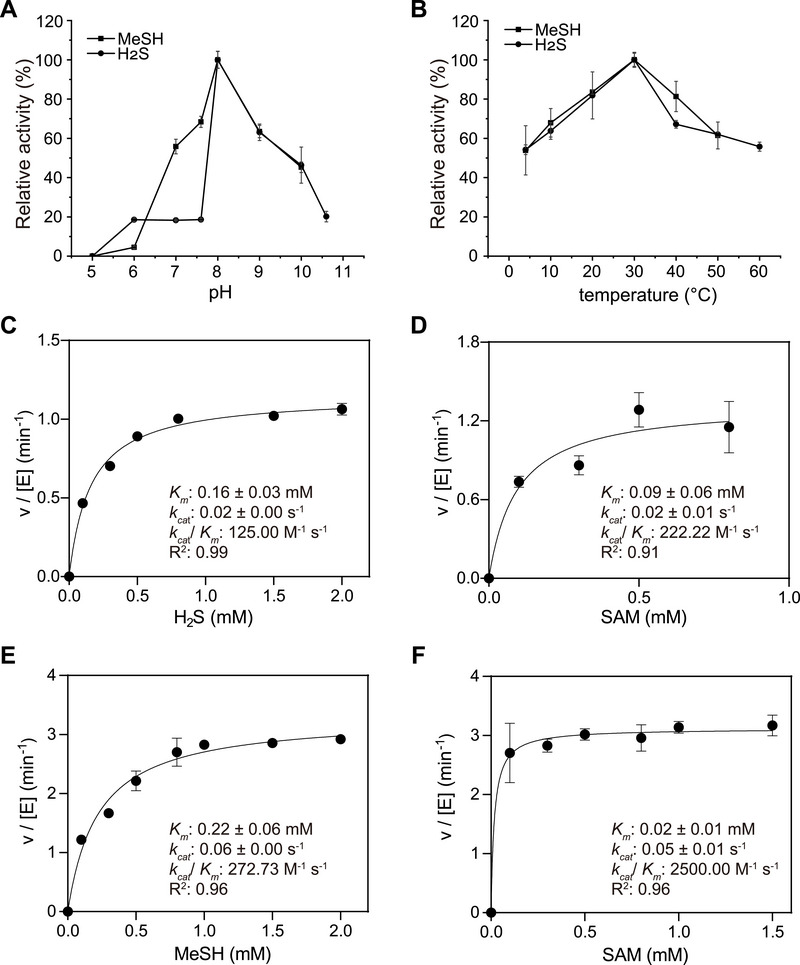
Kinetic characterization of recombinant *^Tb^* MddM2. Effect of pH (A) and temperature (B) on the enzymatic activity of *^Tb^* MddM2. The 100% activity values were 44.73 and 58.73 nmol mg protein^−1^ min^−1^ for MeSH and H_2_S, respectively, at optimum pH, and 35.79 and 29.53 nmol mg protein^−1^ min^−1^ at optimum temperature. Substrate‐dependence of *^Tb^* MddM2 catalytic activity with varying H_2_S concentration (C), or SAM (D) when using H_2_S as a co‐substrate. Substrate‐dependence of *^Tb^* MddM2 catalytic activity with varying MeSH concentration (E), or SAM (F) when using MeSH as a co‐substrate. The kinetic parameters were obtained with 2 µg *^Tb^* MddM2 at pH 8 and 30 °C. Kinetic constants reported in the data panels were obtained by non‐linear fitting of data using the Michaelis–Menten equation as described in Figure 3. The values for DMS production are shown as mean ± s.d. for three biological replicates.

Since *M. poriferae* ZYF656 MddM2 proved problematic to purify, the functionally verified *^Tb^* MddM2 from *Thermobispora bispora* was selected for further analysis. Recombinant GST‐tagged MddM1 and *^Tb^* MddM2 proteins were overexpressed in *E. coli* BL21 (DE3), and proteins of the expected molecular weight were purified (Figure S5A,B, Supporting Information). Both proteins exhibited in vitro SAM‐dependent *S*‐methylation activity with either H_2_S or MeSH to yield DMS. The optimal pH and temperature for MeSH *S*‐methylation were 9 for MddM1 (**Figure** **3**A) and 40 °C (Figure 3B), respectively, whilst its optimal pH and temperature for H_2_S were 7.6 (Figure 3A) and 30 °C (Figure 3B), respectively. *^Tb^* MddM2 showed an optimum pH of 8 (**Figure** **4**A) and temperature of 30 °C for both substrates (Figure 4B). Notably, MddM1 and *^Tb^* MddM2 showed no *S*‐methylation activity toward most other tested sulfur compounds including Coenzyme A (CoA), cysteine (L‐Cys), glutathione (GSH), 2‐mercaptoethanesulfonate (Coenzyme M), or the DMSP synthesis intermediates Met and 4‐methylthio‐2‐hydroxybutyrate (MTHB) (Figure S5C,D, Supporting Information). Thiols, like ethanethiol and 1‐propanethiol, could also serve as the substrates of MddM1 and *^Tb^* MddM2, as was previously shown for MddH (Figure S5C,D, Supporting Information).^[^ ^15^ ^]^

Enzyme kinetics studies showed MddM1 to have a ≈1.6‐fold higher *K_m_* value for MeSH (0.76 mm) than for H_2_S (0.46 mm), and *k_cat_* values that were approximately fivefold higher for MeSH (0.21 s^−1^) (Figure 3C,E). Therefore, MddM1 was approximately threefold more efficient at *S*‐methylating MeSH (*k_cat_*/*K_m_*: 274.33 m ^−1^ s^−1^) than H_2_S (*k_cat_*/*K_m_*: 90.3 m ^−1^ s^−1^), indicating a higher consumption rate of MeSH over its production rate in *vitro*. *^Tb^* MddM2 exhibited comparable *K*_m_ values for H_2_S (0.16 mm) and MeSH (0.22 mm), which were considerably lower than for MddM1 (Figure 4C,E). Conversely, the *k*_cat_ values for *^Tb^* MddM2 were approximately threefold higher for MeSH than H_2_S (Figure 4). Thus, *^Tb^* MddM2 also demonstrated greater efficiency in *S*‐methylating MeSH (*k*_cat_/*K*_m_ ≈ 272.73 m ^−1^ s^−1^) than H_2_S (*k*_cat_/*K*_m _≈ 125 M^−1^ s^−1^). Specific activity data showed MddM1 and *^Tb^* MddM2 exhibited similar activities toward MeSH, which were ≈1.5‐fold lower than for H_2_S, with *^Tb^* MddM2 having slightly higher activity. The MddM1 and *^Tb^* MddM2 specific activities were substantially higher than for MddA,^[^ ^14^ ^]^ but lower than MddH.^[^ ^15^ ^]^

### 2.5 Potential Roles of MddM1 and MddM2 Enzymes

Previous studies implied that the Mdd‐driven conversion of toxic H_2_S and MeSH to non‐toxic DMS may be a cellular strategy for detoxifying these gases and had shown *mddA* and *mddH* transcription to be enhanced by H_2_S and/or MeSH.^[^ ^14^, ^15^ ^]^ Here, *M. poriferae* ZYF656 *mddM1* and *mddM2* transcription was also significantly enhanced by the addition of Met, MeSH, and H_2_S. Induction by Met implied that the bacterial Mdd pathways can efficiently remove excess MeSH released from Met via Met‐gamma‐lyase enzymes when this amino acid is in excess. Note, transcriptional induction was always more prominent for *mddM2* (Figure 1E). Interestingly, *mddM1* and *mddM2* transcription was also enhanced by the addition of H_2_O_2_ to mimic oxidative stress (Figure S6, Supporting Information), something not previously linked to H_2_S and MeSH *S*‐methylation.

**Figure 5:**
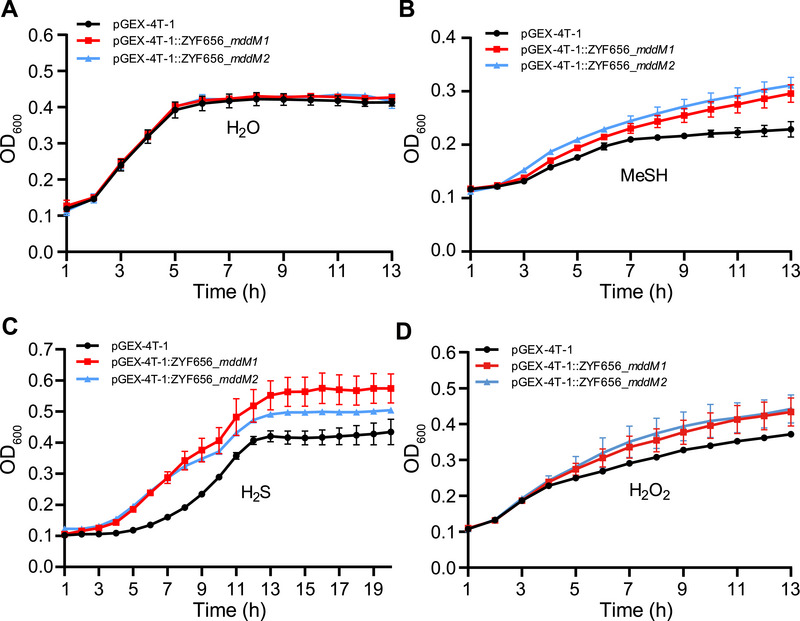
The impact of MddM1 and MddM2 on *E. coli* growth in response to H_2_S, MeSH, and oxidative stress. A) Growth of *E. coli* strains amended with H_2_O (control) in M9 media. B) Growth of *E. coli* strains with 1 mm MeSH in M9 media. C) Growth of *E. coli* strains with 2 mm H_2_O_2_ in M9 media. D) Growth of *E. coli* strains with 1 mm H_2_S in M9 media. Error bars represent the standard deviation from *n *= 3 biological repeats.

The growth of *E. coli* expressing *mddM1* or *mddM2* was examined under stress conditions to study the role of these genes. MddM1 or MddM2 expression in *E. coli* enhanced growth in the presence of H_2_S, MeSH, and H_2_O_2_ (**Figure** **5**), imping these methyltransferases can play a role in detoxification of H_2_S and MeSH, and in oxidative stress protection. For protection against oxidative stress, we hypothesized that DMS might be produced as a source of the antioxidant DMSO,^[^ ^34^ ^]^ as *M. poriferae* ZYF656 contained the trimethylamine monooxygenase gene *tmm*, whose product can oxidize DMS to DMSO.^[^ ^35^ ^]^ Furthermore, *M. poriferae* ZYF656 was able to oxidize DMS, potentially generated from H_2_S and MeSH, to DMSO (7.91 pmol mg^−1^ protein min^−1^). This strain was not able to utilize DMSP, DMSO, MeSH or Met as a sole carbon or sulfur source supporting the role for DMS production in detoxification and/or oxidative stress tolerance and not assimilation.

### 2.6 The Significance of Mdd Systems in Diverse Environments

**Figure 6:**
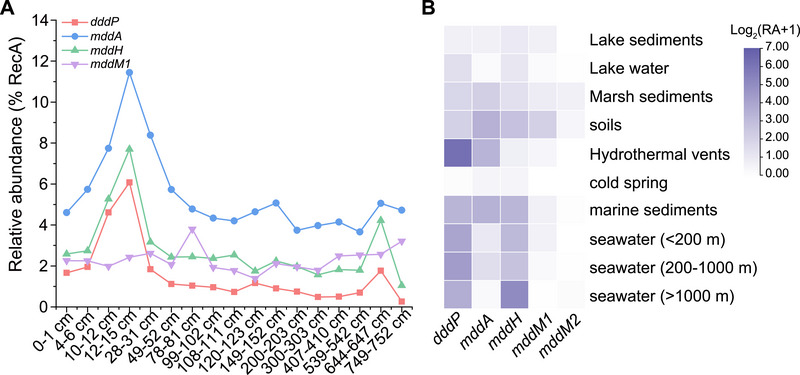
Distribution of *dddP* and *mdd* genes in selected environmental metagenomic datasets. A) Relative abundance of *dddP, mddH, mddA, mddM1*, and *mddM2* in a sectioned Mariana Trench sediment core. B) Comparison of the relative abundance of *dddP, mddH, mddA, mddM1*, and *mddM2* in different environmental metagenomes. The values represent the logarithm to base 2 of their gene abundance plus 1. RA: relative abundance. The numbers of all sequences were normalized to the number of RecA sequences in each metagenome.

To infer the environmental importance of *mddM1*, *mddM2*, *mddA, mddH*, and *dddP*, we assessed their relative gene abundance in diverse environmental multi‐omics data. Focusing initially on Mariana Trench sediments, where H_2_S and MeSH *S*‐methylation was proposed to be important,^[^ ^27^ ^]^ *mddM1* was detected at all depths within sediments from the Trench floor, but no *mddM2* homologues were identified (**Figure** **6**A). The proportion of bacteria with *mddM1* homologues remained relatively stable throughout the gravity column core (1.78–3.8%), with a peak at 78–81 cm (3.8%). *mddM1* was markedly less abundant than *mddA*, the major detected gene in this depth profile, but comparable to *mddH*, except at depths of 10–12 and 12–15 cm (Figure 6A). Surprisingly, the most abundant DMSP lyase gene, *dddP*, was predicted to be present in 0.26–6.07% of bacteria in the Mariana Trench sediments, which was lower than for *mddM1* except in the two samples at 10–12 and 12–15 cm (Figure 6A). These data implied that MddM1 and more prominently other Mdd enzymes may play a more important role in DMS production compared to DMSP lyase pathways in Mariana Trench marine sediments.

Analysis of metagenomes from other diverse environments showed *mddM1* was present in all, but was most abundant in soils (3.3%, Figure 6B), supporting the prediction of H_2_S‐ and MeSH‐dependent DMS production being an important process in sedimentary environments.^[^ ^12^, ^26^, ^36^ ^]^ *mddM1* was predicted in 0.02–0.58% marine bacteria, but its prevalence decreased with increasing seawater depth (Figure 6B). In contrast, the relative abundance of *mddM2* was particularly low in all environments (0–0.58%), and no *mddM2* genes were detected in marine water samples (≤200 m), cold spring, or hydrothermal vents within this dataset (Figure 6B). Cumulatively, the DMSP lyase enzyme DddP was far more abundant than all *mdd* genes in most aquatic samples, but not sediments, implying that DMSP cleavage is the predominant aquatic DMS source (Figure 6B). Moreover, *mddH* also exhibited a wide distribution in various environments (Figure 6B). The taxonomy of prokaryotic *mdd* genes beyond the *Terrabacteria* group and *Proteobacteria* in many metagenomic samples remained challenging. It was worth noting that most MAGs with *mddM1* homologues were annotated as *Actinomycetota*, and 100% of MAGs with *mddM2* homologues were also annotated as *Actinomycetota*, corroborating our above findings that the MddM1 and MddM2 enzymes are primarily in *Actinomycetota*. In Tara Oceans samples from the OM‐RGC marine metagenome database, only two *mddM1* homologous genes were detected at extremely low abundance and exhibited a sparse distribution.^[^ ^37^ ^]^ This low‐level detection of *mddM1* could be due to these actinomycetes being filamentous and filtered out of Tara Oceans samples; or their preference to colonize marine sediments rather than seawater, and the OM‐RGC database comprising largely pelagic samples.

## 3 Discussion

The important gas DMS can be biologically produced from many precursor molecules including H_2_S, MeSH, and DMSO, but DMSP was thought to be the major source. Here, we further highlight the potential importance of H_2_S and MeSH‐dependent DMS production via the discovery and characterization of two novel Mdd enzymes, termed MddM1 and MddM2. MddM1 was exceptionally common in *Actinomycetota* and in terrestrial and marine sediment environments, the latter being known hotspots for DMS production.^[^ ^7^ ^]^

MddM1 and MddM2 encoded SAM‐dependent H_2_S and MeSH methyltransferases which were both taxonomically distinct to the previously identified MddA, MddH, and eukaryotic thiol methyltransferases (Figure 2; Figure S2, Supporting Information).^[^ ^14^, ^15^, ^26^ ^]^ Despite their taxonomic disparity, MddM1, MddM2, MddH, and human TMT1A and TMT1B all shared extended residue similarity, the same conserved SAM‐binding domain, and significant structural similarity (Figure 2; Figure S2, Supporting Information). These data implied that these five *S*‐methyltransferase enzymes may share a common ancestor. In contrast, MddA bore no substantial sequence nor structural similarity to MddM1, MddM2, MddH, TMT1A or TMT1B, implying that it evolved independently. Nevertheless, this study highlighted significant biodiversity in the Mdd enzyme family, which was reminiscent of that observed with DMSP lyases.^[^ ^10^, ^15^, ^38^ ^]^ These data lend further weight to the hypothesis that the enzymatic evolution of H_2_S and MeSH *S*‐methyltransferase and DMSP cleavage activities were far easier than for DMSP demethylation for example.^[^ ^10^, ^15^, ^38^ ^]^

Met, H_2_S, MeSH and likely, MMPA are known to be toxic to cells by inhibiting the mitochondrial electron transport chain.^[^ ^39^ ^]^ These substances can be converted into the harmless gas DMS through a combination of microbial Met‐gamma‐lyase, downstream DMSP demethylation enzymes, and ultimately Mdd‐family *S*‐methyltransferases.^[^ ^14^, ^15^ ^]^ Our data reinforces the hypothesis that microbes with an Mdd enzyme, *S*‐methylate H_2_S and MeSH (potentially from MMPA and/or Met degradation) to produce DMS as a cellular detoxification strategy. In addition, oxidative stress is a continuous factor that bacteria must cope with and a key mechanism associated with MeSH‐induced cellular damage.^[^ ^40^ ^]^ Here, we provide the first evidence for Mdd pathways protecting against oxidative stress through the host upregulating *mdd* genes in response to oxidative stress (from H_2_O_2_ addition), and the action Mdd proteins in its amelioration. Note, cells have many methods to deal with oxidative stress, for example, superoxide dismutase and catalase production, but the specific antioxidant mechanisms of Mdd enzymes remain unknown. It was interesting that there were both membrane‐associated (MddA and MddM2) and cytosolic Mdd (MddH and MddM1) enzymes, perhaps implying extracellular or intracellular sources of the toxic Mdd substrates. A membranous system might better detoxify exterior molecules and vice versa for the cellular‐generated substrates.

Based on the identification of Mdd‐like proteins in the MMETSP database and the subsequent finding that one candidate algal Mdd enzyme lacked *S*‐methyltransferase activity against the canonical substrates, it was possible that these proteins comprise a clade of Mdd‐like methyltransferase enzymes that evolved to methylate a substrate distinct from H_2_S or MeSH, and not necessarily related to sulfur cycling. As noted above, it is also possible that the chosen candidate algal Mdd‐like protein did not properly fold when expressed in *E. coli* or that it required a missing co‐factor for activity. Further studies are required on more representative proteins from this diverse clade to draw robust conclusions on their role and environmental importance. Nevertheless, this finding highlights the critical importance of coupling genomic predictions with functional analyzes and of the necessity of comprehensively considering potential substrate range.

The significance of the Mdd pathways in global DMS production, with far‐reaching implications for atmospheric chemistry, climate, and sulfur cycling, has been underestimated in the past. Here, we showed that the genetic potential for DMS production via Mdd systems could be more prominent in the terrestrial and marine sediments than DMSP‐dependent systems, including those of the deep ocean where *M. poriferae* ZYF656, the source of MddM1 and MddM2, was isolated. It is likely that Met, MeSH, and H_2_S are more prominent in microoxic‐anoxic sediments than in seawater, perhaps explaining the abundance of *mdd* genes, particularly from *Actinomycetota*, in these environments. This study builds on previous identification of Mdd systems to further highlight the growing potential importance of Mdd and DMSP systems to global DMS production in both marine and terrestrial settings.

There is a pressing need for more environmental measurements of MeSH, H_2_S, DMSP, and other potential DMS sources, with DMSP‐ and Mdd‐dependent DMS production rates and multi‐omics (ideally metatranscriptomics and metaproteomics) to allow a better understanding of the relative importance of the microbes and pathways that generate DMS in diverse environments. Without such comprehensive studies, it is very difficult to estimate the exact contribution of Mdd pathways, or any other pathway, to the global DMS production. Nevertheless, this study emphasizes that Mdd systems cannot automatically be ignored as insignificant contributors to global DMS production and introduces the *Actinomycetota* as potentially important contributors in diverse environments.

## 4 Conclusion

This study identified two novel SAM‐dependent methyltransferases, MddM1 and MddM2, predominantly found in diverse marine and terrestrial actinomycetes, many of which were not previously thought to *S*‐methylate H_2_S and MeSH. Thus, it highlighted the large biodiversity in Mdd enzymes, with four distinct known Mdd enzymes, and implied that Mdd enzymes, like DMSP lyases, evolved multiple times. We confirmed the important role of Mdd proteins to detoxify cytotoxic H_2_S and MeSH through their *S*‐methylation yielding DMS, but also, for the first time, oxidative stress amelioration. Given over 50% of *Actinomycetota* contain *mddM1* and the abundance of *mdd* genes in diverse soils and marsh sediments, this study implicates H_2_S/MeSH‐dependent *S*‐methylation as a major and previously underestimated contributor to global DMS production and sulfur cycling. Future research must combine detailed multiomics studies with measurements of Mdd pathways and other DMS production and consumption processes to further elucidate the relative importance of these pathways in diverse marine and terrestrial environments.

## 5 Experimental Section

### Bacterial Strains, Plasmids, and Culture Media

Strains and plasmids used in this study are listed in Table S2 (Supporting Information). *M*. *poriferae* ZYF656 was grown in 2216E complete medium (per liter seawater: 1 g yeast extract, 5 g peptone, 0.01 g ferric phosphate, 20 g agar, pH 7.6) or MBM minimal medium with a mixed carbon source^[^ ^41^ ^]^ at 28 °C, 170 r.p.m. for 24 h. *E. coli* was cultured in LB complete medium or M9 minimal medium at 37 °C overnight. The composition of the M9 minimal medium was as follows (per 200 mL water): 40 mL 5 × M9 salt (per liter water: 64 g Na_2_HPO_4_·7H_2_O, 15 g KH_2_PO_4_, and 2.5 g NaCl), 200 µL 0.1 m CaCl_2_, 400 µL 1 m MgSO_4_, 200 µL 30 mg mL^−1^ thiamine, and 360 µL 50% glycerol. *S. venezuelae* wild‐type and mutant strains were cultured in MYM complete medium (per liter water: 4 g maltose, 4 g yeast extract, 10 g malt extract, 18 g agar, pH 7.3) or MM minimal medium (per liter water: 0.5 g *L*‐asparagine, 0.5 g K_2_HPO_4_, 0.2 g MgSO_4_·7H_2_O, 0.01 g FeSO_4_·7H_2_O, 10 g glucose, 10 g agar, pH to 7–7.2) at 28 °C, 170 r.p.m. for 48 h. Throughout this study, “water” refers to purified, deionized water used consistently for all media preparations. When required for selection, antibiotics were added at the following concentrations: ampicillin (50 µg mL^−1^), chloramphenicol (25 µg mL^−1^), kanamycin (50 µg mL^−1^).

### Isolation of *M. poriferae* ZYF656

*M*. *poriferae* ZYF656 was isolated from 9600 m depth seawater of the Challenger Deep of the Mariana Trench (11°20.605′N, 142°19.557′E), aboard the R/V *Dong Fang Hong 2* on September, 2016. For the isolation of bacteria, a seawater sample (1 mL) was diluted in a gradient and spread on 2216E on board. All plates were incubated at 28 °C for 5–7 days. Most colonies were picked, purified for three times, and preserved at −80 °C with glycerol (15%, v/v). The bacterial genomic DNA was extracted, and then amplified with the universal primers 27F/1492R for bacterial identification (Table S3, Supporting Information). One isolate ZYF656, identified as *M*. *poriferae*, attracted attention as it showed Mdd activity, but lacked all known *mdd* genes.

### Sole Carbon or Sulfur Source Growth Tests

*M*. *poriferae* ZYF656 cells were harvested, washed with MBM medium three times, and used to inoculate fresh MBM medium lacking a carbon or sulfur source. Met (Sigma Aldrich, USA; 5 mm), DMSP (TCI, Shanghai, China; 5 mm), DMSO (Solarbio, Beijing, China; 2 mm), DMS (Sigma Aldrich, USA; 1 mm), glycerol (Sinopharm, Shanghai, China; 10 mm), MeSH (Sigma Aldrich, USA; 1 mm), glucose (Sinopharm, Shanghai, China; 2 mm), sucrose (Sinopharm, Shanghai, China; 2 mm), sodium succinate (Sigma Aldrich, USA; 2 mm) or sodium pyruvate (Yuanye Bio‐Technology Co., Ltd, Shanghai, China; 2 mm) were added as the sole carbon. Where necessary, Met, DMSP, DMSO, DMS, MeSH, or MgSO_4_ (100 µm, respectively) were added as the sole sulfur source. Cultures were incubated at 28 °C, 170 r.p.m. for 10 days, and cell growth was followed by measuring optical density at 600 nm (OD_600_) using a WFJ 2100 spectrophotometer (Unico, Shanghai, China).

### Analysis of Microbial DMS and MeSH Production

*M*. *poriferae* ZYF656 colonies from fresh agar plates were picked and used to inoculate MBM medium (200 µL) containing Met (0.5 mm), MMPA (Tokyo Chemical Industry Co., Ltd., Tokyo, Japan; 0.5 mm), or MeSH (0.5 mm) in a 2 mL vial, which were incubated at 28 °C, 170 r.p.m. for 24 h. To measure DMS and MeSH production from H_2_S, strains were inoculated into 2216E medium without Fe^3+^ and incubated with H_2_S (0.5 mm) at 28 °C for a further 24 h. The *S. venezuelae* wild‐type and its series of knockout, complemented, and overexpressing strains colonies from fresh MYM agar plates were inoculated into solid slants of MM medium (300 µL) containing the addition of Met (1 mm) or MeSH (1 mm) in 2 mL sealed GC vials and incubated at 28 °C for 48 h. The production of MeSH and DMS was expressed in µmol and nmol, respectively. All experiments were performed with three biological replicates.

Headspace DMS and MeSH levels were directly monitored by gas chromatography (GC) using a flame photometric detector (Agilent 7890A GC fitted with a 7693 autosampler) and a HP‐INNOWax 30 m × 0.320 mm capillary column (Agilent Technologies, J&W Scientific). Culture medium with and without substrate was used as a negative control. Calibration curves were generated as previously described.^[^ ^42^ ^]^ The detection limit for DMS and MeSH was 0.2 and 5 nmol, respectively. Bacterial cells were lysed by ultrasonication (JY92 IIN, Scientz, Ningbo, China) and total protein was quantified by Bradford assays (BioRad, Hemel Hempstead, UK). Experiments were conducted in triplicate and quantitative results were shown as mean ± s.d.

### Analysis of Microbial DMSO Production

*M*. *poriferae* ZYF656 colonies from fresh agar plates were picked and inoculated into MBM medium (200 µL) containing DMS (1 mm) in 2 mL sealed vials and incubated at 28 °C, 170 r.p.m. for 24 h. All traces of DMS were eliminated by opening the lid and heating the reaction vials in an 85 °C water bath for 2 h. Subsequently, vials were sealed immediately after the addition of stannous chloride (100 µL; 880 mm, 16.68 g in 100 mL of 37% HCl), and heated at 55 °C for 90 min. DMS was quantified by GC, as described above.

### Genome Sequencing of *M*. *poriferae* ZYF656

*M*. *poriferae* ZYF656 colonies were provided to the Beijing Genomics institution (BGI; Wuhan, China) who collected performed genomic DNA sequencing. Whole genome sequencing was done using the Illumina Hiseq 4000 sequencer system with a 270 bp paired‐end library and PacBio RSII and a 20 kb library. Reads were assembled using Unicycle, sequencing data correction was performed with Pilon v1.1, and artificial correction of ambiguous sites was carried out with REAPR 1.0. Genome annotation was performed by the RASTtk online service with default settings applied.^[^ ^43^ ^]^ Ratified enzymes involved in DMSP/DMS cycling, shown in Table S4 (Supporting Information), were used as query sequences for BLASTp.

### Construction of *M*. *poriferae* ZYF656 Genomic Library

A genomic library of *M. poriferae* ZYF656 was constructed to identify novel Mdd enzymes. High‐quality *M. poriferae* ZYF656 genomic DNA was partially digested with the restriction endonuclease *Bam*HI, and ligated into *Bam*HI‐digested and dephosphorylated plasmid pUC18. Ligated mixes were transformed into *E. coli* JM109 to form a library with approximately 10000 clones, from which eight clones were randomly selected to determine the library quality and coverage. The eight chosen plasmids were analyzed by restriction digestion with *Bam*HI, and all had 20–30 kbp of cloned DNA with distinct restriction digestion profiles and cloned sequences. To screen for clones conferring MeSH‐dependent DMS production, *E. coli* JM109 transformants were cultured in LB medium with ampicillin (50 µg mL^−1^) at 28 °C, 170 r.p.m. for 12–14 h, and diluted 1/50 into M9 medium containing MeSH (1 mm) and ampicillin (50 µg mL^−1^). The strains were cultured at 37 °C for 24 h, and screened by GC. *E. coli* JM109 with empty pUC18 vector and media only, with or without MeSH, were used as controls. Positive clones producing DMS above the negative controls were sequenced by Sangon Biotech (Shanghai, China).

### General Genetic Manipulations

The primers used in this study are shown in Table S3 (Supporting Information). Bacterial genomic DNA was isolated using a FastPure bacteria DNA isolation mini kit‐Box2 (Vazyme, Nanjing, China). Plasmid purification and gel extraction used an E.Z.N.A. plasmid mini kit I and an E.Z.N.A. gel extraction kit (Omega, Georgia, USA), respectively. Routine restriction digestions and ligations were performed as described in Carrión et al. 2015.^[^ ^26^ ^]^ Plasmids pXMJ19 with *^Tb^mddM2* and *mddM1* were individually transferred to *E. coli* DH5α by transformation. Preparation of competent cells: *C*. RES167 was cultured overnight at 30 °C, and subsequently inoculated into BHIS medium (per 200 mL water: 7.4 g brain heart infusion and 18.2 g sorbitol) until the OD_600_ reached 1.75. The cells were harvested by centrifugation at 5000 *g* for 20 min, resuspended in 20 mL precooled TG buffer (per 100 mL water: 1 mm Tris‐HCl and 12 g 87% glycerol), and repeated this step twice. The TG buffer was then replaced with precooled glycerol (10%) and the procedure performed twice more. Finally, the cells were resuspended in glycerol (10%, 1 mL) and dispensed into aliquots (150 µL) in cooled Eppendorf tubes. Plasmids were transformed into competent *C. glutamicum* RES167 by electroporation as previously described.^[^ ^44^ ^]^ To measure DMS production from MeSH or H_2_S of RES167/pXMJ19‐*mddM1* and RES167/pXMJ19‐*^Tb^mddM2*, these two strains were cultured in 2 mL sealed glass vials into M9 medium (200 µL) supplemented with MeSH (1 mm) or H_2_S (1 mm), glucose (2 mm), and chloramphenicol (25 µg mL^−1^) at 30 °C for 24 h.

Full‐length *mddM1* from *M. poriferae* ZYF656 and *^Tb^mddM2* genes were PCR‐amplified from genomic DNA and individually cloned into the pGEX‐4T‐1 vector (Miaoling Biology, Wuhan, China) for expression of GST‐tagged enzymes in *E. coli* BL21 (DE3). Sequencing of all PCR‐amplified products and plasmids was confirmed by Sangon Biotech Co., Ltd.

### Protein Bioinformatics and Localization

The physical and chemical properties of MddM1 and MddM2 were predicted using protParam (https://web.expasy.org/protparam/).^[^ ^45^ ^]^ CELLO v 2.5 (http://cello.life.nctu.edu.tw/) was used to predict Mdd protein subcellular localization.^[^ ^46^ ^]^ The presence of signal peptides in proteins and their cleavage sites was predicted through the SignalP 5.0 server (https://services.healthtech, dtu.dk/service/SignalP‐5.0).^[^ ^47^ ^]^ The transmembrane helices in proteins were predicted by TMHMM‐2.0 (https://services.healthtech, dtu.dk/service/TMHMM‐2.0).^[^ ^48^ ^]^ The membrane and cytoplasmic proteins of *M. poriferae* ZYF656 were extracted using the Bacterial Membrane Protein/Cytoplasmic Protein Extraction kit (Solarbio, Beijing, China) in accordance with the manufacturer's instructions, yielding membrane proteins (400 mg mL^−1^) and cytoplasmic proteins (11 mg mL^−1^). Given that *M*. *porifera* ZYF656 is a Gram‐positive bacterium, the cell lysis process was extended appropriately, typically for 2 to 4 h. Subsequently, membrane proteins (10 mg) and cytoplasmic proteins (1.2 mg) were each resuspended in Tris‐HCl (pH 8) with MeSH (1 mm) and SAM (1 mm) to a final volume (150 µL), and incubated at 37 °C for 3 h. DMS production was quantified as described above.

### Protein Expression and Purification

Recombinant strains containing pGEX‐4T‐1 were cultured at 37 °C overnight with shaking at 170 r.p.m. in LB medium (5 mL) with ampicillin (50 µg mL^−1^) for positive selection. Afterward, the cultures were inoculated into fresh LB medium and cultivated until OD_600_ reached 0.5. Isopropyl*‐ β‐D*‐thiogalactopyranoside (IPTG) was added to cultures at a final concentration (0.1 mm), which were then further incubated at 16 °C for 14–16 h. Cells were harvested, washed, and resuspended in PBS buffer (140 mm NaCl, 2.7 mm KCl, 10 mm Na_2_HPO_4_, 1.8 mm KH_2_PO_4_, pH 7.3), lysed by sonication using an ultrasonic homogenizer JY92 IIN (Scientz, Ningbo, China), and then centrifugated at 12000 *g* for 10 min. Protein was purified from cell supernatant by Glutathione Sepharose 4B affinity chromatography (GE Healthcare, USA), and the bound proteins were eluted in the elution buffer (50 mm Tris‐HCl, 10 mm reduced glutathione, pH 8). Purified proteins were analyzed by SDS‐PAGE, and stored at −80 °C.

To determine the functionality of MddM1 and MddM2 homologues, candidate MddM1 homologous protein sequences from *Streptomyces* sp. SAJ15 (A0A7M3LRJ1), *Planomonospora sphaerica* (A0A161LM11), *Acidobacteriota bacterium* (MCZ6599416.1), *Acidobacteriota bacterium* (MDE3069982.1), *Mycolicibacterium litorale* (A0A6S6P9F0), *Deltaproteobacteria bacterium* (TMB00698.1), *Dictyobacter kobayashii* (WP_126557608.1), *Streptomyces venezuelae* (F2RA35) and the MddM2 homologous protein sequence from *T. bispora* (D6Y5L2) were obtained from UniproKB (Table S5, Supporting Information), and synthesized by Sangon Biotech (Shanghai, China). They were subcloned into pET‐24a and transformed into *E. coli* BL21 (DE3). The recombinant strains were grown in LB with kanamycin (50 µg mL^−1^) to an OD_600_ of 0.5, and then cultured with IPTG (0.1 mm) at 28 °C, 170 r.p.m. for 3 h. Cells containing overexpressed protein were collected and sonicated as described above. Triplicate sonicated mixture (200 µL) of the supernatants and pellets was incubated with SAM (1 mm) and MeSH (1 mm) or H_2_S (1 mm) for 3 h before quantifying the MeSH and DMS levels and protein concentrations.

### In Vitro Characterization of Mdd Enzymes

The enzymatic activity of MddM1 and *^Tb^* MddM2 was measured by detecting MeSH and DMS production as previously described.^[^ ^14^ ^]^ Reaction mixtures (200 µL) containing Tris‐HCl (20 mm, pH 9), purified protein (1 µg MddM1 or 2 µg *^Tb^* MddM2), SAM (1 mm), and MeSH (1 mm) or H_2_S (1 mm) were incubated at 28 °C for 30 min, and stopped by adding HCl (100 µL, 10%). The MeSH and/or DMS produced was monitored by GC. Enzyme‐deficient reaction mixtures provided negative controls to ensure abiotic DMS formation was absent. To determine the optimal temperature, reaction mixtures were incubated at 4 to 60 °C for 30 min. The optimum pH was conducted at the optimal temperature, and Tris‐HCl was replaced with Britton–Robinson buffer (Boric acid, phosphoric acid, and acetic acid, pH adjusted with NaOH) at pH values of 4 to 10.6. For kinetic parameter assays, *K_m_* and *k_cat_* values were determined by nonlinear regression analysis using purified proteins (1 µg MddM1 or 2 µg *^Tb^* MddM2) and SAM (0–2 mm, fixed at 1 mm for MeSH kinetic work), MeSH or H_2_S (0–2 mm, fixed at 1 mm for SAM kinetic work). The enzymatic activities were also examined under the optimum temperature and pH, and quenched by the addition of HCl (100 µL, 10%). Enzyme activity was calculated by the amount of DMS production when using MeSH as substrate, and by the sum of MeSH and twice DMS production when using H_2_S as substrate. Non‐linear fitting of the data was performed by Graphpad Prism8.

### LC‐MS Analysis

LC‐MS was used to confirm the conversion of SAM to *S*‐adenosyl‐homocysteine (SAH) in enzyme assays with purified protein to verify substrate specificity. LC‐MS was carried out on a QTRAP4500 liquid chromatograph mass spectrometer (SCIEX, Netherlands) with a SunFire C18 reversed‐phase column (4.6 × 250 mm, 5 µm particle size, Waters, United States). The MS spray chamber conditions were as follows: curtain gas 35 psi, ion spray voltage 5000 V, ion spray temperature 450 °C, ion source gas1 40 psi, ion source gas1 45 psi, collision gas medium. Solvent A was ammonium acetate (50 mm, pH 5.5). Solvent B was 20% acetonitrile and 80% solvent A. The samples were eluted with a linear gradient of 95% solvent A to 95% solvent B over 16 min at a flow of 0.8 mL min^−1^. Prior to use, the solvent was subjected to ultrasonic shock for 20 mins to remove dissolved gases. The injection volume was 10 µL. All samples were centrifuged at 12000 *g* for 15 min, after which, each sample (100 µL) was transferred to 2 mL glass vials equipped with internal cannulas. The targeted mass transitions corresponding to [M+H]^+^ of SAH and SAM were detected as previously described.^[^ ^49^, ^50^ ^]^

### Determination of MddM1 and MddM2 Activity in *E. coli*

*E. coli* BL21 strains containing cloned *mddM1* and *mddM2* genes or empty vector were cultured in LB medium (5 mL) containing ampicillin (50 µg mL^−1^) until an OD_600_ of 0.8 was reached. The cells were diluted tenfold into M9 minimal medium (300 µL) with IPTG (0.1 mm) and MeSH (0.5 mm) or H_2_S (0.5 mm), and incubated at 28 °C for 18 h.^[^ ^26^ ^]^ DMS production, MeSH production, and protein concentrations were determined as above.

### Construction of *Streptomyces venezuelae* Mutants

A mutation of *mddM1* (*S. venezuelae* Δ*mddM1*) was constructed in *S. venezuelae* (GenBank accession number: NZ_CP018074.1). gRNA sequences to the target region were ordered as single‐stranded oligos with *Bbs* I overhangs from Integrated DNA technologies (IDT) and annealed at equal molarity in HEPES buffer by heating at 95 °C for 5 min before cooling to 4 °C (at 1 °C s^−1^). To assemble into the pCRISPomyces‐2 vector, Golden Gate reactions were set up using purified backbone (100 ng) and insert (3 µL) in the presence of T4 ligase buffer (2 µL), T4 ligase (1 µL), *Bbs* I (1 µL), and dH_2_O up to a total volume (20 µL). Around 1 kbp of flanking DNA from either side of the target gene was PCR amplified using Q5 DNA polymerase.

pCRISPomyces‐2 containing the gRNA of interest was digested with *Xba* I and dephosphorylated with shrimp alkaline phosphatase to prevent re‐ligation. Flanking DNAs were assembled into the digested vector backbones containing the gRNAs using Gibson Assembly. DNAs were incubated in a ratio of 1:3 (plasmid/insert) in the presence of Gibson Assembly master mix (NEB) at 50 °C for 1 h. The resulting reaction mix was then transformed into *E. coli* and plated on selective media at 37 °C overnight, and the resulting colonies were screened by colony PCR.

Confirmed plasmids were transformed into *E. coli* ET12567 containing pUZ8002. These were then grown overnight at 37 °C, 200 r.p.m., sub‐cultured 1:20 in fresh LB + relevant antibiotics, and grown to OD_600_ 0.4–0.6. Pellets from culture (10 mL) were washed twice with fresh LB to remove antibiotics. Meanwhile, *S. venezuelae* spores (200 µL) were pre‐germinated in 2 × YT (500 µL; per liter water: 16 g tryptone, 10 g yeast extract, 5 g NaCl, pH 7) at 52 °C for 10 min. The two cell types were mixed, resuspended in fresh LB, and plated onto SFM medium (per liter water: 20 g soy flour, 20 g mannitol, 20 g agar) containing MgCl_2_ (10 mm) and incubated at room temperature for 16 h. For selection of desired ex‐conjugants, Nalidixic acid (0.5 mg) and selection antibiotic was added in dH_2_O (1 mL) to each plate, and cultures returned to the 30 °C incubator for 2–3 days or until colonies appeared.

For overexpression, pIJ10257 containing the constitutive ErmE* promoter, was digested with *Nde* I and *Hin*d III and gel purified as described above. The gene of interest was PCR amplified using Q5 Polymerase, and plasmids were assembled using Gibson assembly. Confirmed plasmids were transformed into *E. coli* ET12567 containing pUZ8002 for conjugation into both wild‐type *S. venezuelae* or its *mddM1* mutant. Resulting exconjugants were confirmed to contain the overexpression plasmid by antibiotic selection and colony PCR using Biotaq red. All *Streptomyces* strains were routinely grown on MYM medium at 30 °C for 3 days until the green spore pigment was visible indicating a complete life cycle. The plasmids and primers used are listed in Tables S2 and S3 (Supporting Information).

### Growth Analyzes of *E. coli* with *mddM1* and *mddM2* Genes

*E. coli* BL21 (DE3) strain containing *mddM1* gene, *mddM2* gene, or empty vector was cultured in LB medium (5 mL) at 37 °C overnight, and the OD_600_ was adjusted to 0.5. The cells were then diluted tenfold into fresh M9 minimal medium with MeSH (1 mm), H_2_S (1 mm), or H_2_O_2_ (2 mm) and ampicillin (50 µg mL^−1^) in a 96‐well microplate, and incubated at 37 °C.^[^ ^14^ ^]^ The absorbance of the bacterial suspensions was measured at 600 nm using a Multiskan GO microplate reader (Thermo scientific, USA). An equal amount of distilled water was added to the bacterial suspensions instead of MeSH, H_2_S, or H_2_O_2_ as the negative control.

### RT‐qPCR Analysis

*M. poriferae* ZYF656 was cultured in 2216E medium at 28 °C until logarithmic growth phase was reached. Cells were diluted tenfold into fresh MBM minimal medium containing Met (1 mm), H_2_S (1 mm), or MeSH (1 mm) and incubated at 28 °C, 170 r.p.m. for 8 h. Cells cultured in MBM medium without substrate were used as controls. Each sample was performed in triplicate, and collected by centrifugation at 5000 *g* for 10 min. Total RNA was extracted using a RNeasy Mini kit (Qiagen, Germany), and reverse transcribed using an NZY First‐Strand cDNA Synthesis kit (Nzytech, China). Fluorescence quantitative PCR was performed using a QuantStudio 5 System (Thermo Fisher Scientific, USA). Data were analyzed by the 2^−^ *^△△CT^* method, and *recA* was used as an internal standard. Forward and reverse primer pairs were designed as shown in Table S3 (Supporting Information).

### Bioinformatics Analysis

BLASTp was used to identify candidate MddM1 and MddM2 homologues (identity ≥ 40%, *E*‐value ≤ e‐30, and coverage ≥ 70%) in the Uniprot (https://www.uniprot.org/) and NCBI NR (https://www.ncbi.nlm.nih.gov/) databases.^[^ ^51^ ^]^ MddM1 and MddM2 of *M. poriferae* ZYF656 were used as the query sequences, respectively. The phylogenetic tree was constructed at the IQ‐TREE website (http://iqtree.cibiv.univie.ac.at/). The information for representative MddM proteins is shown in Table S6 (Supporting Information). The amino acid sequences were aligned using MAFFT (https://mafft.cbrc.jp/alignment/server/) and uploaded to the Gblocks website (http://molevol.cmima.csic.es/castresana/Gblocks server) for gap removal. The maximum‐likelihood method and the LG+F+G4 model of amino acid substitution were used with 1000 bootstrap replications. The tree was visualized by Chiplot.^[^ ^52^ ^]^ MddM1 homologous proteins shown in Table S4 (Supporting Information) from distinct branches or bacterial clades were selected to further study to verify their Mdd activities.

To explore the distribution of MddM1, MddM2, MddA, and MddH across the *Actinomycetota*, ratified protein sequences of MddM1, MddM2, MddA, and MddH were used as query sequences to perform Hidden Markov Model (HMM)‐based searches (*E‐*value ≤ e‐55) from all representative genomes of *Actinomycetota* from NCBI (42815 in total) using HMMER (v3.4) (https://github.com/EddyRivasLab/hmmer.git), BLASTp searches against peptide databases from the genomes obtained in the previous step, and set the alignment threshold to identity ≥ 40% and coverage ≥ 70%. Metagenomes containing actinomycetes with at least 5% abundance were screened from the Sandpiper 0.2.0 (http://sandpiper.qut.edu.au), and the biogeographic distribution of the actinomycetes was plotted by R (v. 4.0.3) using scatterpie and ggplot2.

To analyze the distribution of MddM1, MddM2, MddA, MddH, and DddP in different environmental metagenomes, environmental metagenomes were downloaded using the online webserver from the integrated microbial genomes and microbiomes (IMG/M) system^[^ ^53^ ^]^ and NCBI SRA database,^[^ ^54^ ^]^ and the genome information is shown in Table S7 (Supporting Information). Metagenomic sequencing and binning were performed as previously described.^[^ ^27^ ^]^ The MddM1, MddM2, MddA, MddH, and DddP sequences used for the metagenome analysis are detailed in Table S4 (Supporting Information). RecA sequences were extracted from Cheng et al. 2023.^[^ ^27^ ^]^ HMM‐based searches (*E‐*value ≤ e‐55) for Mdd homologues in metagenomes from different environments using cutoff values of identity ≥ 40%, *E‐*value ≤ e‐55, and coverage ≥ 70% were retrieved by BLASTp. The abundance of *mdd* genes was calculated using the percentages of bacterial harboring genes normalized to the single‐copy housekeeping gene *recA*.

For Eukaryotes, homologues of MddM1, MddM2, and MddH were identified from re‐assemblies of the MMETSP database (https://doi.org/10.5281/zenodo.740440) using HMM searches with an *E*‐value of 1 × 10^−30^ for MddM1 and MddM2, and 1 × 10^−80^ for MddH.^[^ ^33^ ^]^ The obtained sequences were further refined with an identity ≥ 35% and coverage ≥ 70%. The maximum‐likelihood tree of eukaryotic Mdd homologues was constructed using IQ‐TREE. The resulting tree was visualized in Itol.^[^ ^55^ ^]^

## Conflict of Interest

The authors declare no conflict of interest.

## Author Contributions

R.G., Z.G., Y.Z., and Y.H.Z. contributed equally to this work. X.‐H.Z. and J.D.T. were responsible for the conceptualization of the study. Experiments were carried out by R.G. and Y.Z. (identification and characterization of MddM, genomic library construction and growth experiments), Z.G. (H_2_S experiments, candidate MddM expression and characterization, mutant strains construction, membrane protein extraction), Y.H.Z. (screening and identification of novel Mdd strains, establishment of Mdd activity experimental method), R.D. (mutant construction), C.S. (phenotypic experiments of the mutant strains) and Y.F.Z. (isolation of bacterial strains). Data analysis was carried out by R.G. (genetic taxonomy), Z.G. (genome and MMETSP database alignment), H.C. (metagenome), R.L. (bioinformation) and A.J.G. (enzymatic kinetics). X.‐H.Z. and Y.H.Z. were accountable for the supervision of the study. The original draft of the manuscript was written by R.G., Z.G., and Y.Z. Writing the review and editing was also performed by X.‐H.Z., J.D.T., R.G., Z.G., Y.H.Z., and A.J.G.

## Supporting information



Supporting Information

## Data Availability

All data needed to evaluate the conclusions in the paper are present in the paper and/or the Supplementary information.
