# Supplementary material for: Two Novel S‐methyltransferases Confer Dimethylsulfide Production in Actinomycetota
Source: Adv Sci (Weinh). 2025 Dec 3;13(9):e10141. doi: 10.1002/advs.202510141 (PMC12904073; doi:10.1002/advs.202510141)
Supplement: Supplementary file 1 — Supporting Information [file ADVS-13-e10141-s001.docx]

Supporting Information

**Two Novel *S*-methyltransferases Confer Dimethylsulfide Production in *Actinomycetota***

Ruihong Guo, Zihua Guo, Yi Zhou, Yunhui Zhang, Haojin Cheng, Rebecca Devine, Chuang Sun, Ronghua Liu, Yanfen Zheng, Andrew J. Gates, Jonathan D. Todd*, Xiao-Hua Zhang*

**
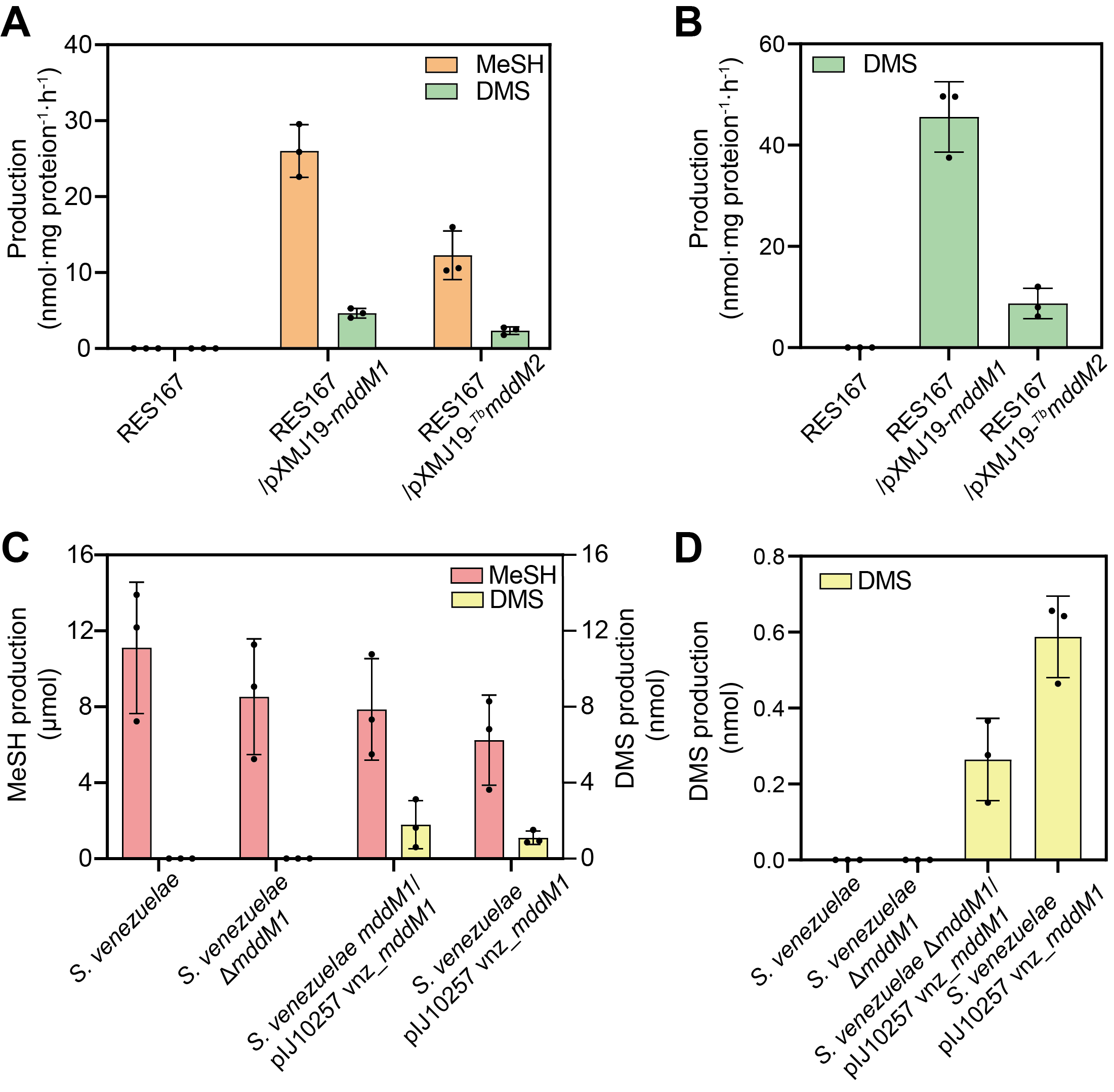
**

**Figure S1.** Gas chromatography detection of DMS and MeSH produced from *Corynebacterium* *glutamicum* RES167, *Streptomyces venezuelae* and their respective mutants. MeSH and DMS production from *C.* *glutamicum* RES167 containing cloned pXMJ19-*mddM1*, pXMJ19-*^Tb^mddM2* or empty vector, when grown with 1 mM H_2_S (A) or MeSH (B). MeSH and DMS production from *S. venezuelae* wild type and its mutant strains with 1 mM Met (C) or 1 mM H_2_S (D). The values for DMS and MeSH production are shown as mean ± s.d.


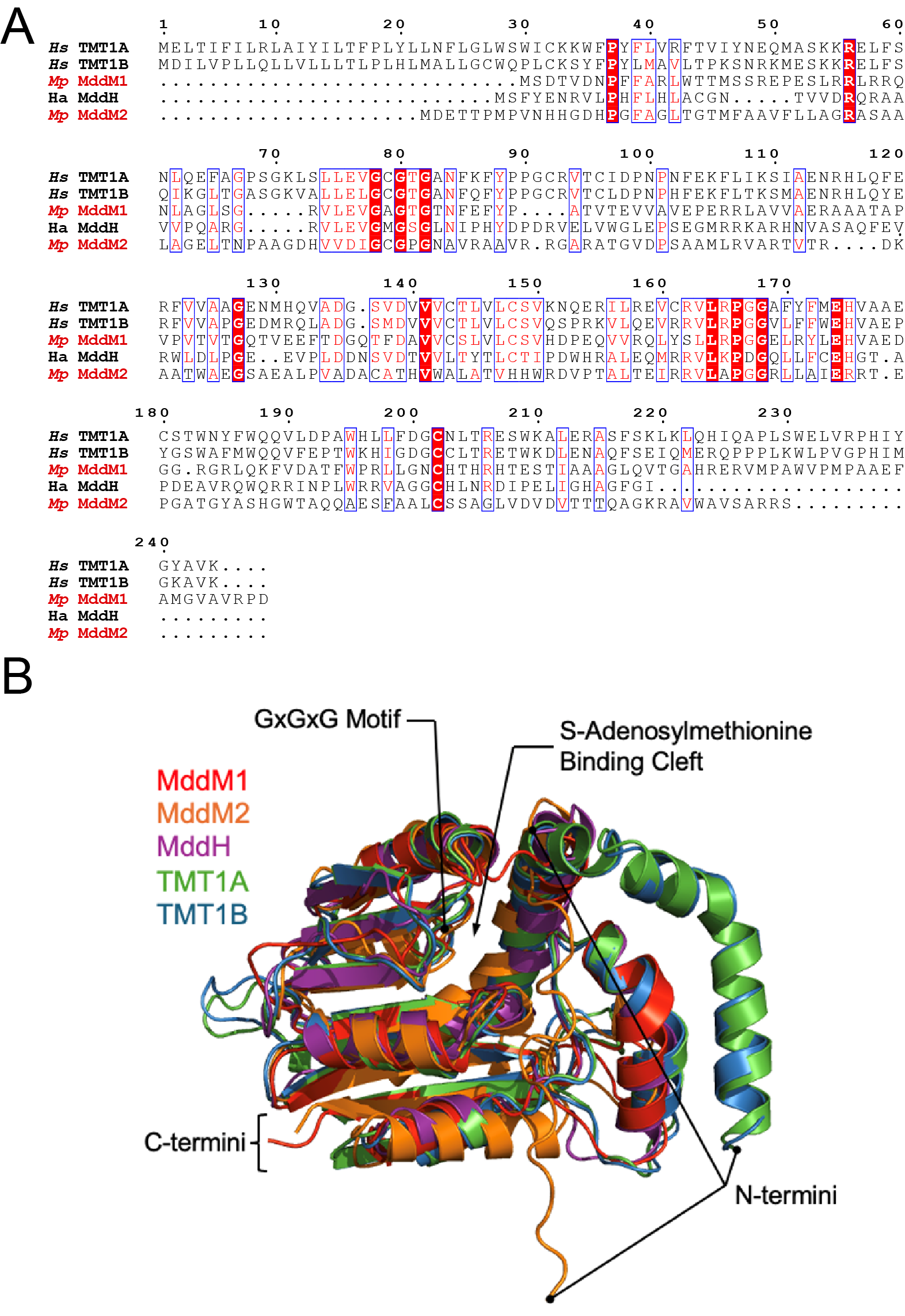


**Figure S2.** Sequence comparison and structural prediction with other putative and known SAM-dependent methyltransferases. A) Multiple sequence alignment using ClustalW 2.1 and ESPript 3.0. The GxGxG motif and SAM binding cleft are indicated by boxes, and fully conserved residues are highlighted in white on a red background. B) Structural prediction for MddM1 (red), MddM2 (orange), MddH (purple), TMT1A (green) and TMT1B (blue) using AlphaFold3, with the GxGxG motif and SAM-binding cleft annotated. Structural image was generated using Pymol ver 3.0.0.


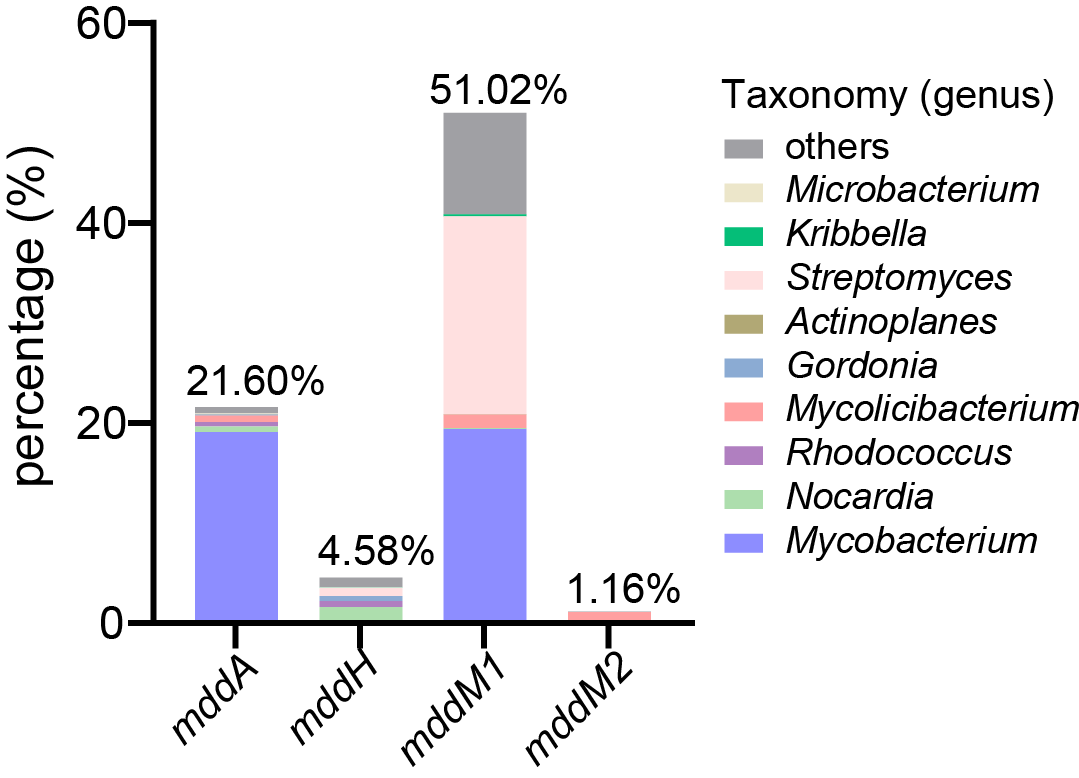


**Figure S3.** Distribution of *mddA*, *mddH*, *mddM1* and *mddM2* genes in all *Actinomycetota* genomes (n=42815) downloaded from NCBI.


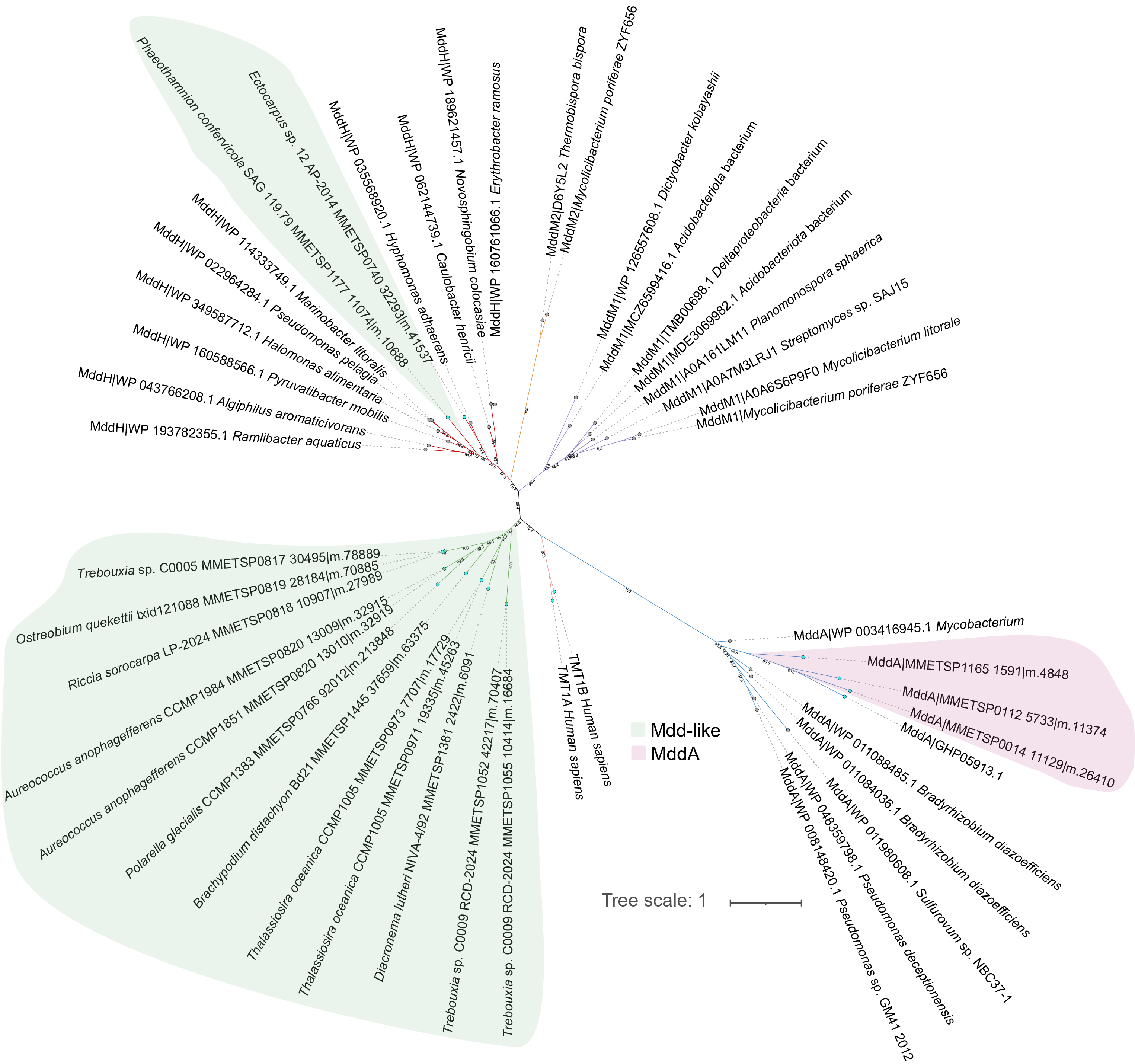


**Figure S4. Maximum-likelihood phylogenetic tree of Mdd proteins in eukaryotes.** Branches are colored according to different Mdd proteins. Functional MddA sequences in eukaryotes (pink shading) are used as reference sequences. Mdd-like sequences (green shading) indicate matched Mdd proteins in eukaryotes. Lines are colored to distinguish different clusters. Blue and gray dots denote sequences from eukaryotic and prokaryotic organisms, respectively. The scale bar indicates 1 amino acid substitutions per site.

**
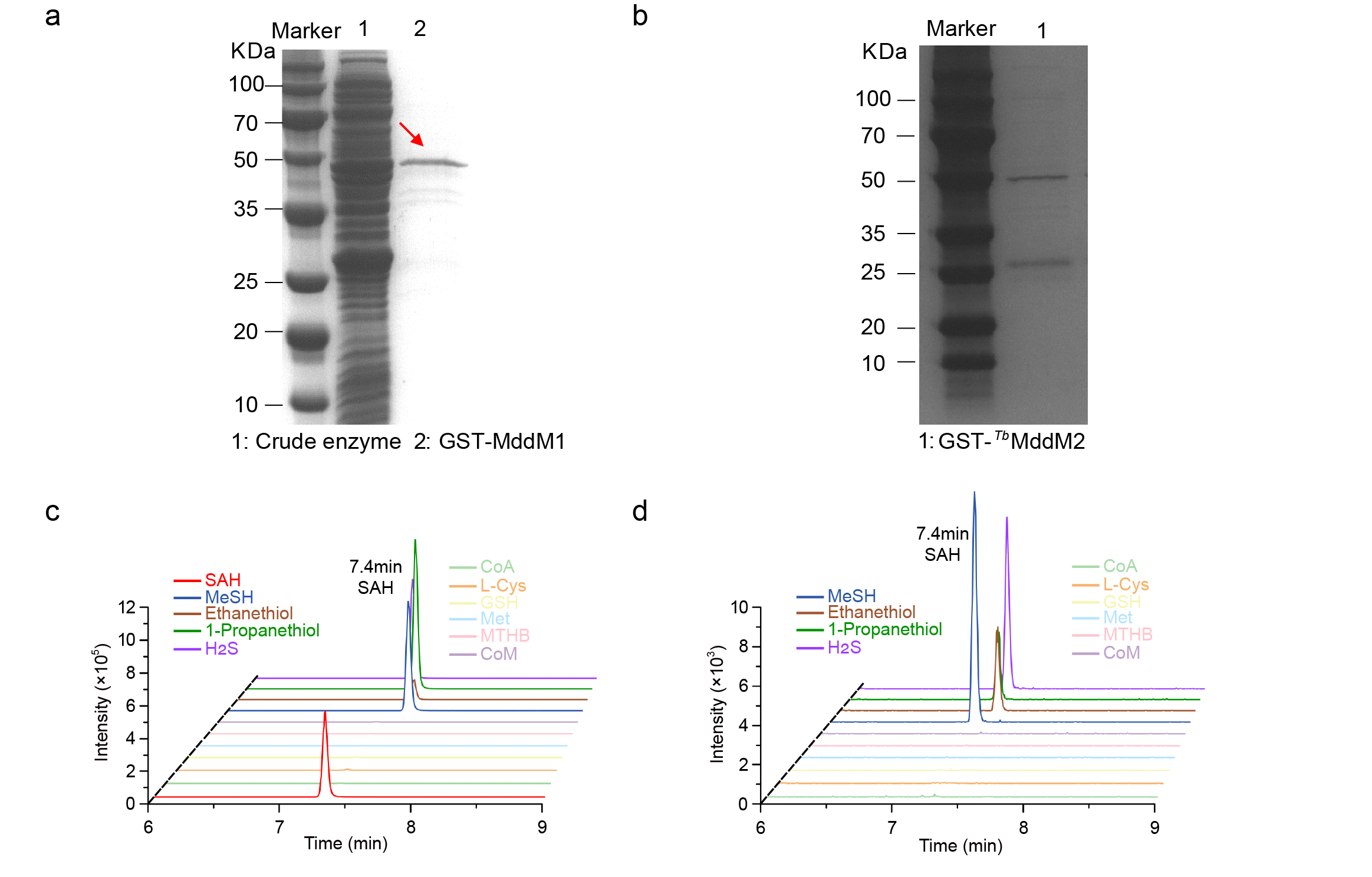
**

**Figure S5. Characterization of the recombinant MddM1 and *^Tb^*MddM2.** A) Properties of purified recombinant GST-tagged MddM1 from *M. poriferae* ZYF656 run on a 12% precast SDS-PAGE gel. Lanes: Marker, prestained protein ladder. Lane 1, lysate before purification; lane 2, purified protein (molecular weight: 50.02 kDa). B) Purified recombinant GST-tagged MddM2 of *Thermobispora bispora*. Lanes: Marker, prestained protein ladder. Lane 1, purified protein (molecular weight: 48.65 kDa). The larger band corresponds to the uncleaved protein, while the smaller band represents the processed form. The ability of MddM1 (C) and *^Tb^*MddM2 (D) to *S*-methylate a range of substrates (as detailed) as monitored by the formation of *S*-adenosyl-homocysteine (SAH) from *S*-adenosyl-methionine (SAM). MeSH, methanethiol; H_2_S, Hydrogen sulfde; CoA, coenzyme A; *L*-Cys, cysteine; GSH, glutathione; Met, methionine; MTHB, 4-methylthio-2-hydroxybutyrate; CoM, Coenzyme M.


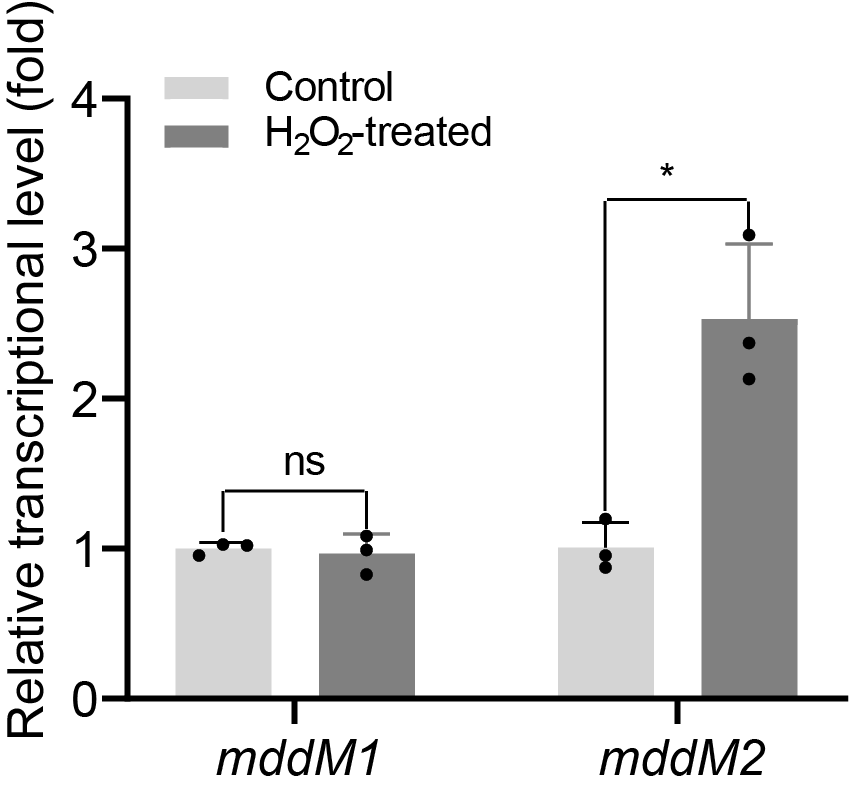


**Figure S6. RT-qPCR analyzes of *mddM1* and *mddM2* genes of *M. poriferae* ZYF656 in the presence of 2 mM H_2_O_2_.** The values are shown as mean ± s.d. for three biological replicates. Significance was determined by Student’s *t*-test (ns, not significant; **p*<0.05).

**Table S1. DMS cycling gene analysis of the *M.* *poriferae* ZYF656 genome by BLASTp against functional gene list.**

| ZYF656 gene ID | Clostest homologues | Homologue accession ID | Amio acid identity (%) | Evalue | Coverage (%) |
| --- | --- | --- | --- | --- | --- |
| PP625016 | AcuH | AAV93475.1 | 64 | 5.05e-117 | 99 |
| PP661496 | Tmm | ACK52489.1 | 61 | 0 | 100 |
| PP661497 | DmdB | WP_011047771.1 | 51 | 0 | 99 |
| PP661492 | MegL | AAO46884.1 | 42 | 5.77e-100 | 84 |

**Table S2. Strains and plasmids involved in this study.**

| Strains or plasmids | Description | Reference or source |
| --- | --- | --- |
| *Mycolicibacterium poriferae* ZYF656 | Wild-type isolate; Available from Zhang lab | This study;  Zhang Lab |
| *Escherichia coli* JM109 | recA1 endA1 gyrA96 thi-1 hsdR17 supE44 Δ(lac-proAB/F) [traD36 proAB^+^ lacIq lacZΔM15] | ^[1]^ |
| *E. coli* DH5α | Transformed cells for gene cloning | AngYu Biotechnologies (Shanghai, China) |
| *E. coli* BL21 (DE3) | Transformed cells for gene expression | AngYu Biotechnologies (Shanghai, China) |
| pUC18 | Plasmid vectors for genomic library in *E. coli* DH5α, ampicillin-resistant | This study;  Zhang Lab |
| pET-24a | Plasmid vector for cloning gene in *E.* coli DH5α, kanamycin-resistant | This study;  Zhang Lab |
| pUCm-T | Plasmid vector for cloning gene in *E. coli* DH5α, ampicillin-resistant | Sangon Biotech |
| pGEX-4T-1 | Plasmid vector for cloning gene in *E. coli* DH5α, ampicillin-resistant | Miaoling Biology |
| pXMJ19 | Plasmid vector for gene expression, chloramphenicol-resistant | This study;  Zhang Lab |
| *E. coli* NEB5a | fhuA2 Δ(argF-lacZ)U169 phoA glnV44 Φ80 Δ(lacZ) M15 gyrA96 recA1 relA1 endA1 thi-1 hsdR17 | New England Biolabs |
| *E. coli* ET12567 | dam^-^ dcm^-^ hsdS^-^ | ^[2]^ |
| pCRISPomyces-2 | oriT, reppSG5(ts), oriColE1, sSpcas9,  synthetic guide RNA cassette, ampicillin-resistant | ^[3]^ |
| pUZ8002 | RK2 derivative with a mutation in oriT, kanamycin-resistant | ^[4]^ |
| pIJ10257 | oriT, ΦBT1attB-int, ermEp*, pMS81 backbone, hygromycin B-resistant | ^[5]^ |
| pIJ10257 vnz_mddM1 | For the deletion of vnz_mddM1 | This study; Jon. Lab |
| pCRISPomyces-2 vnz_mddM1 | For the overexpression of vnz_mddM1 | This study; Jon. Lab |
| pXMJ19::RES167_ *mddM1* | pXMJ19 containing the *mddM1* gene of ZYF656 | This study;  Zhang Lab |
| pXMJ19::RES167_ *^Tb^mddM1* | pXMJ19 containing the *mddM2* gene of *Thermobispora bispora* | This study;  Zhang Lab |
| pGEX-4T-1::ZYF656*_mddM1* | pGEX-4T-1 containing the *mddM1* gene of ZYF656 | This study;  Zhang Lab |
| pGEX-4T-1::ZYF656*_mddM2* | pGEX-4T-1 containing the *mddM2* gene of ZYF656 | This study;  Zhang Lab |
| pGEX-4T-1::Tb_*MddM2* | pGEX-4T-1 containing the *mddM2* gene of *Thermobispora bispora* | This study; |
|  |  | Zhang Lab |
| *S. Venezuelae* Δ*mddM1* | knockout strains | This study; Jon. Lab |
| *S. venezuelae*/pIJ10257 vnz_*mddM1* | overexpressed strains | This study; Jon. Lab |
| *S. venezuelae* ΔmddM1/pIJ10257 vnz_*mddM1* | complementary strains | This study; Jon. Lab |

**Table S3. Primers used in this study.**

| Gene name | Primer sequences (5’ - 3’) | Function and reference |
| --- | --- | --- |
| 27F | AGAGTTTGATCCTGGCTCAG | the universal primers for bacterial identification. |
| 1492R | GGTTACCTTGTTACGACTT |  |
| *mddM1*-pGEX4T1-BamHI-F | gatctggttccgcgtggatccATGAGCGACACAGTCGATAATCC | PCR amplification of *mddM1* from ZYF656 and cloning into pGEX-4T-1 |
| *mddM1*-pGEX4T1-EcoRI-R | ctcgagtcgacccgggaattcTCAGTCCGGCCGCACGGC |  |
| *mddM2*-pGEX4T1-BamHI-F | gatctggttccgcgtggatccATGGACGAAACCACACCGATG | PCR amplification of *mddM2* from ZYF656 and cloning into pGEX-4T-1 |
| *mddM2*-pGEX4T1- EcoRI-R | ctcgagtcgacccgggaattcTCAGGACCGGCGCGCGCT |  |
| *^Tb^mddM2*-pGEX4T1-BamHI-F | gatctggttccgcgtggatccATGAGCGCGGCGGACGTT | PCR amplification of *mddM2* from *Thermobispora bispora* and cloning into pGEX-4T-1 |
| *^Tb^mddM2*-pGEX4T1- EcoRI-R | ctcgagtcgacccgggaattcCGGACGGGTCGCGGTAAC |  |
| *mddM1*-pXMJ19-BamHI-F | caggtcgactctagaggatccATGAGCGACACAGTCGATAATCC | PCR amplification of *mddM1* from ZYF656 and cloning into pXMJ19 |
| *mddM1*-pXMJ19-EcoRI-R | caaaacagccaagctgaattcTCAGTCCGGCCGCACGGC |  |
| *^Tb^mddM2*-pXMJ19-BamHI-F | caggtcgactctagaggatccATGAGCGCGGCGGACGTT | PCR amplification of *mddM2* from *Thermobispora bispora* and cloning into pXMJ19 |
| *^Tb^mddM2*-pXMJ19- EcoRI-R | caaaacagccaagctgaattcCGGACGGGTCGCGGTAAC |  |
| ZYF656_*mddM1*-F | tgaggagttcaccgacggt | RT-qPCR amplification of *mddM1* |
| ZYF656_*mddM1*-R | aaggtcgcgtcgacgaactt |  |
| ZYF656_*mddM2*-F | accacgtcgtcgacatcg | RT-qPCR amplification of *mddM2* |
| ZYF656_*mddM2*-R | tcgccaatgcccagacgt |  |
| ZYF656_*recA*-F | cagttcctgcagttcaccgt | RT-qPCR amplification of *recA* |
| ZYF656_*recA*-R | tcacgcagattgggtgacag |  |
| vnz_*mddM1* KO-1F | gctcggttgccgccgggcgttttttaTCTAGAGCTCCGCGAGAGAAGGACACC | *mddM1* deletion flank in *Streptomyces venezuelae* |
| vnz_*mddM1* KO-1R | GCTGCTGCGACCAGGCGAGCTCGCCGGATGGTGGACACGGGACG | *mddM1* deletion flank in *S. venezuelae* |
| vnz_*mddM1* KO-2F | GCGAGCTCGCCTGGTCGCAGCAGCCCGTCGTCGCCGTGCGTCC | *mddM1* deletion flank in *S. venezuelae* |
| vnz_*mddM1* KO-2R | gcaacgcggcctttttacggttcctggccTCTAGACGGTGCCGATGACGAGCGC | *mddM1* deletion flank in *S. venezuelae* |
| vnz_*mddM1* gRNA-F | acgcGGCACGTCGAACGCCCGGTA | *mddM1* deletion gRNA in *S. venezuelae* |
| vnz_*mddM1* gRNA-R | aaacTACCGGGCGTTCGACGTGCC | *mddM1* deletion gRNA in *S. venezuelae* |
| vnz_*mddM1* test-F | CGTGACGAGCGACGACGACC | *mddM1* test primers in *S. venezuelae* |
| vnz_*mddM1* test-R | GGACGCACGGCGACGACGG | *mddM1* test primers in *S. venezuelae* |
| vnz_*mddM1* test INT-F | GGAGGGCAAGACGGTGAAGAACC | *mddM1* test primers in *S. venezuelae* |
| vnz_*mddM1* test INT-R | CCTTCCAGAAGGCCAGCACG | *mddM1* test primers in *S. venezuelae* |
| *mddM1* pIJ10257-F | gtctagaacaggaggccccatatgCAGACGACCGGGCTGAGGAG | *mddM1* overexpression in *S. venezuelae* |
| *mddM1* pIJ10257-R | ctcatgagaacctaggatccaagcttCGTATCTGAAGATCGGTCATGGCC | *mddM1* overexpression in *S. venezuelae* |

**Table S4. Accession numbers of the functional ratified enzymes involved in DMS cycling.**

| Protein | Organism | Accession number |
| --- | --- | --- |
| MddM1 | *Mycolicibacterium poriferae* ZYF656 | PP661493 |
|  | *Streptomyces* sp. SAJ15 | A0A7M3LRJ1 |
|  | *Streptomyces venezuelae* | F2RA35 |
|  | *Dictyobacter kobayashii* | WP_126557608.1 |
|  | *Deltaproteobacteria bacterium* | TMB00698.1 |
|  | *Mycolicibacterium litorale* | A0A6S6P9F0 |
|  | *Acidobacteriota bacterium* | MDE3069982.1 |
|  | *Acidobacteriota bacterium* | MCZ6599416.1 |
|  | *Planomonospora sphaerica* | A0A161LM11 |
| MddM2 | *Mycolicibacterium poriferae* ZYF656 | PP661494 |
|  | *Thermobispora bispora* | D6Y5L2 |
| MddA  (EC 2.1.1.334) | *Pseudomonas deceptionensis* | WP_048359798.1 |
|  | *Mycobacterium tuberculosis* H37Rv | NP_217755.1 |
|  | *Bradyrhizobium diazoefficiens* USDA 110 Blr1218 | NP_767858.1 |
|  | *Bradyrhizobium diazoefficiens* USDA 110 Blr5741 | NP_772381.1 |
|  | *Cyanothece* sp. ATCC 51142 | YP_001803274.1 |
|  | *Bradyrhizobium* sp. YR681 | WP_008143861.1 |
|  | *Pseudomonas* sp. GM41 | WP_008148420.1 |
|  | *Crocosphaera chwakensis* | WP_008274188.1 |
|  | *Pseudomonas fragi* | WP_010655917.1 |
|  | *Mycobacterium intracellulare* | OBH46854.1 |
|  | *Neptunicoccus sediminis* | WP_069301345.1 |
|  | *Haladaptatus* sp. W1 | WP_217493340.1 |
|  | *Haladaptatus* sp. PSR5 | WP_227374427.1 |
|  | *Pycnococcus provasolii* | GHP05913.1 |
|  | *Chrysocystis fragilis* CCMP3189 | MMETSP1165_Transcript_1591\|m.4848 |
|  | *Nitzschia* sp. RCC80 | MMETSP0014_Transcript_11129\|m.26410 |
|  | *Lotharella globose* CCCM811 | MMETSP0112_Transcript_5733\|m.11374 |
| MddH  (EC 2.1.1.-) | *Algiphilus aromaticivorans* DG1253 | WP_043766208.1 |
|  | *Marinobacter litoralis* Sw-45 | WP_114333749.1 |
|  | *Pseudomonas pelagia* CL-AP6 | WP_022964284.1 |
|  | *Hyphomonas adhaerens* MHS-3 | WP_035568920.1 |
|  | *Pyruvatibacter mobilis* CGMCC_1.15125 | WP_160588566.1 |
|  | *Novosphingobium colocasiae* KCTC 32255 | WP_189621457.1 |
|  | *Erythrobacter ramosus* DSM 8510 | WP_160761066.1 |
|  | *Ramlibacter aquaticus* LMG 30558 | WP_193782355.1 |
|  | *Caulobacter henricii* CB4 | WP_062144739.1 |
|  | *Halomonas alimentaria* EF61 | WP_013333065.1 |
| DSYB  (EC 2.1.1.373) | *Prymnesium parvum* CCAP946/6 | - |
|  | *Chrysochromulina tobin* CCMP291 | KOO32714 |
|  | *Lingulodinium polyedrum* CCMP1936 | - |
|  | *Alexandrium tamarense* ATSP1-B | - |
|  | *Acropora cervicornis* | - |
|  | *Fragilariopsis cylindrus* CCMP1102 | OEU17621 |
|  | *Symbiodinium microadriaticum* CCMP2467 | OLQ07620 |
| TpMMT  (EC 2.1.1.67) | *Thalassiosira pseudonana* CCMP1335 | Tp23128 |
| DsyB  (EC 2.1.1.373) | *Labrenzia aggregata* IAM 12614 | WP_006937642 |
|  | *Labrenzia aggregate* LZB033 | WP_075282486 |
|  | *Pseduooceanicola batsensis* HTCC2597 | WP_009805585 |
|  | *Pelagibaca bermudensis* HTCC2601 | WP_007801186 |
|  | *Sediminimonas qiaohouensis* DSM 21189 | WP_026756701 |
|  | *Thalassobaculum salexigens* DSM 19539 | WP_084618911 |
|  | *Sagittula stellate* E-37 | WP_005854984 |
|  | *Amorphus coralli* DSM 19760 | WP_018697905 |
| MmtN  (EC 2.1.1.-) | *Novosphingobium* sp. MBES04 | WP_052321947 |
|  | *Croceicoccus mobilis* | WP_066775518 |
|  | *Thalassospira* sp. HJ | WP_044830103 |
|  | *Thalassospira* sp. MCCC_1A01148 | WP_062957385 |
|  | *Thalassospira indica* | WP_064788038 |
|  | *Thalassospira tepidiphila* MCCC_1A03514 | WP_064780488 |
|  | *Thalassospira australica* | WP_033070178 |
|  | *Thalassospira lucentensis* | WP_022734010 |
|  | *Thalassospira* sp. MCCC_1A02898 | WP_063085993 |
|  | *Thalassospira profundimaris* sp. DSM17430 | WP_008888945 |
|  | *Labrenzia* sp. OB1 | WP_068409229 |
|  | *Roseovarius indicus* 01 | WP_064261696 |
|  | *Roseovarius indicus* 02 | WP_057814729 |
|  | *Roseovarius indicus* 03 | KRS18724.1 |
|  | *Rhodobacter aestuarii* | WP_076485456 |
|  | *Saccharothrix syringae* | WP_033429235 |
|  | *Micromonospora nigra* | WP_091090849 |
|  | *Agrobacterium vitis* | WP_071204336 |
|  | *Nocardiopsis chromatogenes* | WP_017624909 |
|  | *Streptomyces mobaraensis* NBRC_13819 | EME99407 |
| DmdA  (EC 2.1.1.269) | *Ruegeria pomeroyi* DSS-3 | AAV95190 |
|  | *Pelagibacter ubique* HTCC1062 | WP_011281570 |
|  | *Dinoroseobacter shibae* DFL 12 | WP_012178987 |
|  | *marine gammaproteobacterium* HTCC2080 | WP_007233625 |
|  | *Candidatus Pelagibacter* sp. HTCC7211 | WP_008546106 |
|  | *Candidatus Puniceispirillum marinum* IMCC1322 | WP_013044947 |
| DddD  (EC 2.8.3.-) | *Marinomonas* sp. MWYL1 | ABR72937 |
|  | *Oceanimonas doudoroffii* DSM 7028 | AEQ39135 |
|  | *Psychrobacter* sp. J466 | ACY02894 |
|  | *Halomonas* sp. HTNK1 | ACV84065 |
|  | *Burkholderia ambifaria* AMMD | WP_011659284 |
|  | *Pseudomonas* sp. J465 | ACY01992 |
| DddL  (EC 4.4.1.3) | *Sulfitobacter* sp. EE-36 | ADK55772 |
|  | *Rhodobacter_sphaeroides* | WP_011336734 |
|  | *Rhodobacter sphaeroides* 2.4.1 | YP_351475 |
|  | *Fulvimarina_pelagi* | WP_007067665 |
|  | *Loktanella_vestfoldensis* | WP_019955302 |
|  | *Pseudooceanicola_batsensis* | WP_009805827 |
|  | *Labrenzia aggregata* LZB033 | AKS25183 |
|  | *Labrenzia aggregate* LZD062 | KP639183 |
| DddP  (EC 3.4.-.-) | *Roseovarius nubinhibens* ISM | EAP77700 |
|  | *Ruegeria pomeroyi* DSS-3 | WP_044029245 |
|  | *Oceanimonas doudoroffii* DSM 7028 | AEQ39091 |
|  | *Oceanimonas doudoroffii* DSM 7028 | AEQ39103 |
|  | *Fusarium graminearum* PH-1 | XP_389272 |
| DddQ  (EC 4.4.1.3) | *Ruegeria pomeroyi* DSS-3 | WP_011047333 |
|  | *Roseovarius nubinhibens* ISM | EAP76002 |
|  | *Roseovarius nubinhibens* ISM | EAP76001 |
|  | *Ruegeria lacuscaerulensis* ITI-1157 | WP_005978225 |
|  | GOS databases | ECW91654 |
|  | GOS databases | EBP74803 |
|  | GOS databases | ECX82089 |
| DddW  (EC 4.4.1.3) | *Ruegeria pomeroyi* DSS-3 | AAV93771 |
|  | *Roseobacter* sp. MED193 | EAQ44306.1 |
| DddY  (EC 4.4.1.3) | *Alcaligenes faecalis* M3A | ADT64689 |
|  | *Shewanella putrefaciens* CN-32 | ABP77243 |
|  | *Desulfovibrio acrylicus* | SHJ73420 |
|  | *Ferrimonas kyonanensis* DSM 18153 | WP_028114584 |
|  | *Acinetobacter bereziniae* | ENV21217 |
| DddK  (-) | *Candidatus Pelagibacter ubique* HTCC1062 | AAZ21215 |
|  | *Alphaproteobacterium* HIMB5 | AFS47241 |
|  | *Candidatus Pelagibacter ubique* HTCC9022 | WP_028037226 |
|  | *Pelagibacteraceae bacterium* BACL20 MAG-120920-bin64 | KRP06000 |
|  | *Candidatus Pelagibacter ubique* | WP_006997514 |
|  | *Candidatus Pelagibacter ubique* | WP_027306832 |
|  | *Candidatus Pelagibacter ubique* | WP_018413735 |
| Almal  (EC 4.4.1.3) | *Emiliania huxleyi* CCMP1516 | XP_005784450 |
|  | *Emiliania huxleyi* CCMP1516 | XP_005763983 |
|  | - | sp\|P0DN22 |
| DddX  (-) | *Psychrobacter* sp. (56811) | 7CM9_1 |
|  | *Psychrobacter* sp. P11G5 | WP_068035783 |
|  | *Sporosarcina* sp. P33 | WP_081242855 |
|  | *Roseobacteraceae* | WP_109384856 |
|  | *Marinobacterium jannaschii* | WP_084332639 |
| DddU  (-) | *Ruegeria faecimaris* DSM 28009 | WP_142638590.1 |
|  | *Phaeobacter inhibens* P66 | WP_058277181.1 |
|  | *Aliiroseovarius pelagivivens* KCTC 42459 | WP_108856353.1 |
|  | *Pseudaestuariivita atlantica* MCCC 1A09432 | WP_050532617.1 |
|  | *Amylibacter cionae* H-12 | WP_188671731.1 |
|  | *Shimia sediminis* ZQ172 | WP_127114513.1 |
|  | *Leisingera aquimarina* DSM 24565 | WP_027260001.1 |
| AcuH  (EC 4.2.1.17) | *Ruegeria lacuscaerulensis* ITI-1157 | EEX08788.1 |
|  | *Ruegeria pomeroyi* DSS-3 | AAV93475.1 |
| DmdB  (EC 6.2.1.44) | *Ruegeria pomeroyi* | WP_011047771 |
|  | *Ruegeria pomeroyi* | WP_011046428 |
|  | *Candidatus Pelagibacter ubiqu* | WP_011281571 |
| DmdC  (EC 1.3.99.41) | *Ruegeria pomeroyi* | WP_011049476 |
|  | *Burkholderia thailandensis* | WP_009892931 |
|  | *Ruegeria lacuscaerulensis* ITI-1157 | EEX10128 |
|  | *Ruegeria pomeroyi* | WP_011048615 |
|  | *Pseudomonas* | WP_003114720 |
|  | *Pseudomonas* | WP_003114561 |
|  | *Burkholderia thailandensis* | WP_009889880 |
|  | *Ruegeria lacuscaerulensis* ITI-1157 | EEX08676 |
| DmdD  (EC 4.2.1.155) | *Ruegeria pomeroyi* DSS-3 | Q5LLW6.1 |
| DorA (-) | *Rhodobacter Capsulatus* | 1DMR_A |
| DsoB  (EC 1.14.13.245) | *Acinetobacter sp.* | BAA23331.1 |
| Tmm  (EC 1.14.13.148) | *Ruegeria pomeroyi* DSS-3 | AAV94838.1 |
|  | *Methylophaga aminisulfidivorans* | WP_007144064 |
|  | *Methylocella silvestris* BL2 | ACK52489.1 |
|  | *Roseovarius* sp. 217 | EAQ26624.1 |
|  | *Candidatus Pelagibacter ubique* HTCC1002 | EAS85405.1 |
|  | *Candidatus Pelagibacter* sp. HTCC7211 | EDZ59919.1 |
| DdhA  (EC 1.8.5.3) | *Sagittula stellata* E-37 | EBA07058.1 |
|  | *Rhodovulum sulfidophilum* | AAN46632.1 |
| DmoA  (EC 1.14.13.131) | *Hyphomicrobium sulfonivorans* | 6AK1_A |
| MTO  (EC 1.8.3.4) | *Hyphomicrobium* sp. | ATJ26742.1 |
|  | *Methylophaga thiooxydans* | WP_008290534 |
|  | *Ruegeria pomeroyi* | WP_011242048 |
|  | *Hyphomicrobium denitrificans* ATCC 51888 | ADJ22562.1 |
|  | *Pseudovibrio ascidiaceicola* | WP_093522951 |
|  | *Methylococcus capsulatus str. Bath* | AAU90430.1 |

**Table S5. List of candidates MddM proteins with their accession numbers.**

| Organism and enzymes | Candidate protein | Accession number | Length  (aa) | Identity (%) | *E*-value | Coverage  (%) |
| --- | --- | --- | --- | --- | --- | --- |
| *Streptomyces* sp. SAJ15 | MddM1 | A0A7M3LRJ1 | 224 | 47 | 2e-58 | 98 |
| *Planomonospora sphaerica* | MddM1 | A0A161LM11 | 219 | 48 | 8e-44 | 97 |
| *Acidobacteriota bacterium* | MddM1 | MCZ6599416.1 | 220 | 42.23 | 1e-30 | 95 |
| *Acidobacteriota bacterium* | MddM1 | MDE3069982.1 | 216 | 43.6 | 3e-39 | 99 |
| *Mycolicibacterium litorale* | MddM1 | A0A6S6P9F0 | 209 | 68.4 | 7.41e-94 | 100 |
| *Deltaproteobacteria bacterium* | MddM1 | TMB00698.1 | 216 | 47.47 | 8e-49 | 99 |
| *Dictyobacter kobayashii* | MddM1 | WP_126557608.1 | 213 | 41.12 | 3e-31 | 90 |
| *Streptomyces venezuelae* | MddM1 | F2RA35 | 221 | 47.4 | 4.21e-45 | 94 |
| *Thermobispora_bispora* | MddM2 | D6Y5L2 | 204 | 58.6 | 8.4e-80 | 99 |

**Table S6. Protein sequences used in the molecular phylogenetic analysis of MddM proteins.**

| Strain | Accession number | Identity (%) | *E*-value | Coverage (%) |
| --- | --- | --- | --- | --- |
| *Mycolicibacterium llatzerense* | [A0A0D1LHS4](https://www.uniprot.org/uniprotkb/A0A0D1LHS4/entry) | 66 | 7.17e-90 | 98 |
| *Mycolicibacterium chubuense* | [A0A0J6WP30](https://www.uniprot.org/uniprotkb/A0A0J6WP30/entry) | 77.5 | 8.45e-110 | 97 |
| *Streptomyces caatingaensis* | [A0A0K9XKP9](https://www.uniprot.org/uniprotkb/A0A0K9XKP9/entry) | 46 | 4.15e-44 | 98 |
| *Streptomyces acidiscabies* | [A0A0L0KEK7](https://www.uniprot.org/uniprotkb/A0A0L0KEK7/entry) | 45.5 | 4.05e-43 | 96 |
| *Mycolicibacterium fortuitum* | [A0A0N9YFI5](https://www.uniprot.org/uniprotkb/A0A0N9YFI5/entry) | 70.9 | 7.80e-100 | 99 |
| *Streptomyces* sp. | [A0A0U3N707](https://www.uniprot.org/uniprotkb/A0A0U3N707/entry) | 42.9 | 4.13e-44 | 94 |
| *Streptomyces kanasensis* | [A0A117IX25](https://www.uniprot.org/uniprotkb/A0A117IX25/entry) | 46.3 | 4.27e-46 | 93 |
| *Streptomyces longwoodensis* | [A0A117QQW5](https://www.uniprot.org/uniprotkb/A0A117QQW5/entry) | 44.5 | 4.06e-43 | 93 |
| *Streptomyces regalis* | [A0A124G714](https://www.uniprot.org/uniprotkb/A0A124G714/entry) | 43 | 3.95e-41 | 92 |
| *Mycolicibacterium wolinskyi* | [A0A132PKZ9](https://www.uniprot.org/uniprotkb/A0A132PKZ9/entry) | 73.7 | 7.89e-101 | 96 |
| *Planomonospora sphaerica* | [A0A161LM11](https://www.uniprot.org/uniprotkb/A0A161LM11/entry) | 48.1 | 3.99e-42 | 93 |
| *Micromonospora siamensis* | [A0A1C5HYN6](https://www.uniprot.org/uniprotkb/A0A1C5HYN6/entry) | 49 | 4.48e-49 | 98 |
| *Micromonospora inositola* | [A0A1C5K1J4](https://www.uniprot.org/uniprotkb/A0A1C5K1J4/entry) | 49 | 4.37e-48 | 99 |
| *Streptomyces rubrolavendulae* | [A0A1D8FX15](https://www.uniprot.org/uniprotkb/A0A1D8FX15/entry) | 44.5 | 4.12e-44 | 93 |
| *Mycobacterium holsaticum* | [A0A1E3RYM9](https://www.uniprot.org/uniprotkb/A0A1E3RYM9/entry) | 72.2 | 7.87e-101 | 100 |
| *Streptomyces agglomeratus* | [A0A1E5PFA8](https://www.uniprot.org/uniprotkb/A0A1E5PFA8/entry) | 44.3 | 3.99e-42 | 91 |
| *Mycobacterium grossiae* | [A0A1E8Q0C4](https://www.uniprot.org/uniprotkb/A0A1E8Q0C4/entry) | 70.7 | 7.38e-93 | 95 |
| *Streptomyces indicus* | [A0A1G8ZYS7](https://www.uniprot.org/uniprotkb/A0A1G8ZYS7/entry) | 43.1 | 4.12e-44 | 96 |
| *Thermomonospora echinospora* | [A0A1H5TMW6](https://www.uniprot.org/uniprotkb/A0A1H5TMW6/entry) | 44.9 | 4.13e-44 | 96 |
| *Streptomyces radiopugnans* | [A0A1H9DTD7](https://www.uniprot.org/uniprotkb/A0A1H9DTD7/entry) | 45.5 | 3.97e-42 | 96 |
| *Streptomyces aidingensis* | [A0A1I1J6R3](https://www.uniprot.org/uniprotkb/A0A1I1J6R3/entry) | 44.7 | 4.10e-44 | 95 |
| *Actinomadura madurae* | [A0A1I5JN64](https://www.uniprot.org/uniprotkb/A0A1I5JN64/entry) | 44.9 | 4.11e-44 | 89 |
| *Amycolatopsis arida* | [A0A1I6ACS4](https://www.uniprot.org/uniprotkb/A0A1I6ACS4/entry) | 42.9 | 3.86e-40 | 99 |
| *Cryptosporangium aurantiacum* | [A0A1M7PAZ4](https://www.uniprot.org/uniprotkb/A0A1M7PAZ4/entry) | 47.8 | 4.48e-49 | 97 |
| *Streptomyces* sp. | [A0A1Q5L9W3](https://www.uniprot.org/uniprotkb/A0A1Q5L9W3/entry) | 46.2 | 4.27e-46 | 94 |
| *Frankia soli* | [A0A1S1Q4Q4](https://www.uniprot.org/uniprotkb/A0A1S1Q4Q4/entry) | 46.7 | 4.24e-46 | 95 |
| *Mycolicibacterium fallax* | [A0A1X1QXP6](https://www.uniprot.org/uniprotkb/A0A1X1QXP6/entry) | 66.7 | 7.15e-90 | 99 |
| *Mycobacterium celatum* | [A0A1X1RHM3](https://www.uniprot.org/uniprotkb/A0A1X1RHM3/entry) | 68.9 | 7.40e-94 | 98 |
| *Mycobacterium doricum* | [A0A1X1T427](https://www.uniprot.org/uniprotkb/A0A1X1T427/entry) | 68.9 | 7.62e-97 | 99 |
| *Mycobacterium dioxanotrophicus* | [A0A1Y0C0J6](https://www.uniprot.org/uniprotkb/A0A1Y0C0J6/entry) | 68.3 | 7.53e-96 | 97 |
| *Streptomyces alboflavus* | [A0A1Z1W640](https://www.uniprot.org/uniprotkb/A0A1Z1W640/entry) | 43.3 | 3.92e-41 | 92 |
| *Streptomyces albireticuli* | [A0A1Z2LAT5](https://www.uniprot.org/uniprotkb/A0A1Z2LAT5/entry) | 44.3 | 4.21e-45 | 92 |
| *Plantactinospora* sp. | [A0A248YMX7](https://www.uniprot.org/uniprotkb/A0A248YMX7/entry) | 50.7 | 4.50e-50 | 98 |
| *Streptomyces* sp. | [A0A2B8AU63](https://www.uniprot.org/uniprotkb/A0A2B8AU63/entry) | 45.9 | 4.27e-46 | 95 |
| *Streptomyces cinnamoneus* | [A0A2G1XLL5](https://www.uniprot.org/uniprotkb/A0A2G1XLL5/entry) | 46 | 4.13e-44 | 92 |
| *Streptomyces* sp. | [A0A2G9DWW0](https://www.uniprot.org/uniprotkb/A0A2G9DWW0/entry) | 43.1 | 3.99e-42 | 96 |
| *Streptomyces carminius* | [A0A2M8MC47](https://www.uniprot.org/uniprotkb/A0A2M8MC47/entry) | 50 | 4.34e-47 | 93 |
| *Streptomyces lunaelactis* | [A0A2R4SY05](https://www.uniprot.org/uniprotkb/A0A2R4SY05/entry) | 42.7 | 3.95e-41 | 94 |
| *Streptomyces tirandamycinicus* | [A0A2S1SSA7](https://www.uniprot.org/uniprotkb/A0A2S1SSA7/entry) | 44.5 | 3.93e-41 | 93 |
| *Streptomyces solincola* | [A0A2S9PP34](https://www.uniprot.org/uniprotkb/A0A2S9PP34/entry) | 45.5 | 4.24e-46 | 99 |
| *Actinoplanes italicus* | [A0A2T0KFI6](https://www.uniprot.org/uniprotkb/A0A2T0KFI6/entry) | 46.9 | 4.31e-47 | 94 |
| *Micromonospora* sp. | [A0A317DKQ9](https://www.uniprot.org/uniprotkb/A0A317DKQ9/entry) | 48.3 | 4.41e-48 | 99 |
| *Streptomyces armeniacus* | [A0A345XTR3](https://www.uniprot.org/uniprotkb/A0A345XTR3/entry) | 46 | 4.38e-48 | 96 |
| *Geodermatophilus* sp. | [A0A366ZGR4](https://www.uniprot.org/uniprotkb/A0A366ZGR4/entry) | 48.3 | 4.41e-48 | 96 |
| *Blastococcus* sp. | [A0A367AEG0](https://www.uniprot.org/uniprotkb/A0A367AEG0/entry) | 50.2 | 4.40e-48 | 95 |
| *Geodermatophilus* sp. | [A0A372J006](https://www.uniprot.org/uniprotkb/A0A372J006/entry) | 54.6 | 4.55e-51 | 95 |
| *Mycolicibacterium tokaiense* | [A0A378TMP4](https://www.uniprot.org/uniprotkb/A0A378TMP4/entry) | 69.8 | 7.19e-91 | 100 |
| *Bailinhaonella thermotolerans* | [A0A3A4B515](https://www.uniprot.org/uniprotkb/A0A3A4B515/entry) | 48.3 | 4.46e-49 | 98 |
| *Streptomyces klenkii* | [A0A3B0BAP6](https://www.uniprot.org/uniprotkb/A0A3B0BAP6/entry) | 46 | 4.16e-44 | 85 |
| *Streptomyces luteoverticillatus* | [A0A3Q9FYC4](https://www.uniprot.org/uniprotkb/A0A3Q9FYC4/entry) | 46.7 | 4.27e-46 | 93 |
| *Streptomyces xinghaiensis* | [A0A3R7J2V2](https://www.uniprot.org/uniprotkb/A0A3R7J2V2/entry) | 44.2 | 4.19e-45 | 93 |
| *Mycolicibacterium aurum* | [A0A3S4S664](https://www.uniprot.org/uniprotkb/A0A3S4S664/entry) | 78.9 | 8.59e-112 | 99 |
| *Streptomyces* sp. | [A0A401MW77](https://www.uniprot.org/uniprotkb/A0A401MW77/entry) | 44.2 | 4.31e-47 | 92 |
| *Streptomyces netropsis* | [A0A445N4Q1](https://www.uniprot.org/uniprotkb/A0A445N4Q1/entry) | 45.8 | 4.26e-46 | 93 |
| *Herbihabitans rhizosphaerae* | [A0A4Q7L474](https://www.uniprot.org/uniprotkb/A0A4Q7L474/entry) | 47.5 | 4.03e-43 | 84 |
| *Streptomyces kasugaensis* | [A0A4Q9HNW5](https://www.uniprot.org/uniprotkb/A0A4Q9HNW5/entry) | 44.5 | 4.07e-43 | 95 |
| *Kribbella turkmenica* | [A0A4R4XDQ2](https://www.uniprot.org/uniprotkb/A0A4R4XDQ2/entry) | 46.2 | 3.98e-42 | 96 |
| *Actinomadura rubrisoli* | [A0A4R5B5Q5](https://www.uniprot.org/uniprotkb/A0A4R5B5Q5/entry) | 46.3 | 4.23e-46 | 99 |
| *Streptomyces gardneri* | [A0A4Y3RE30](https://www.uniprot.org/uniprotkb/A0A4Y3RE30/entry) | 46.4 | 4.07e-43 | 95 |
| *Actinomadura hallensis* | [A0A543IEA8](https://www.uniprot.org/uniprotkb/A0A543IEA8/entry) | 49.8 | 4.19e-45 | 99 |
| *Streptomyces qinzhouensis* | [A0A5B8ICR1](https://www.uniprot.org/uniprotkb/A0A5B8ICR1/entry) | 47.2 | 4.39e-48 | 88 |
| *Baekduia soli* | [A0A5B8TZG5](https://www.uniprot.org/uniprotkb/A0A5B8TZG5/entry) | 49.5 | 3.83e-40 | 88 |
| *Streptomyces alkaliterrae* | [A0A5P0YTG6](https://www.uniprot.org/uniprotkb/A0A5P0YTG6/entry) | 44.7 | 4.31e-47 | 94 |
| *Mycolicibacterium vanbaalenii* | [A0A5S9R976](https://www.uniprot.org/uniprotkb/A0A5S9R976/entry) | 79.4 | 8.85e-116 | 99 |
| *Streptomyces jumonjinensis* | [A0A646KMU2](https://www.uniprot.org/uniprotkb/A0A646KMU2/entry) | 44.5 | 4.28e-46 | 93 |
| *Streptomyces* sp. | [A0A6B2RY25](https://www.uniprot.org/uniprotkb/A0A6B2RY25/entry) | 45 | 4.02e-42 | 93 |
| *Streptomyces taklimakanensis* | [A0A6G2BIV8](https://www.uniprot.org/uniprotkb/A0A6G2BIV8/entry) | 48.1 | 4.15e-44 | 95 |
| *Streptomyces coryli* | [A0A6G4TSL1](https://www.uniprot.org/uniprotkb/A0A6G4TSL1/entry) | 46 | 4.03e-43 | 95 |
| *Saccharopolyspora* sp. | [A0A6H1RBJ1](https://www.uniprot.org/uniprotkb/A0A6H1RBJ1/entry) | 46.1 | 4.08e-43 | 97 |
| *Streptomyces* sp. | [A0A6I4NL08](https://www.uniprot.org/uniprotkb/A0A6I4NL08/entry) | 43.3 | 4.05e-43 | 94 |
| *Phytoactinopolyspora halotolerans* | [A0A6L9SDV5](https://www.uniprot.org/uniprotkb/A0A6L9SDV5/entry) | 47.6 | 4.44e-49 | 99 |
| *Mycolicibacterium poriferae* | [A0A6N4VHZ4](https://www.uniprot.org/uniprotkb/A0A6N4VHZ4/entry) | 97.1 | 1.06e-141 | 100 |
| *Mycolicibacterium litorale* | [A0A6S6P9F0](https://www.uniprot.org/uniprotkb/A0A6S6P9F0/entry) | 68.4 | 7.41e-94 | 100 |
| *Actinomadura verrucosospora* | [A0A7D3ZXH9](https://www.uniprot.org/uniprotkb/A0A7D3ZXH9/entry) | 45.9 | 4.21e-45 | 94 |
| *Nocardia wallacei* | [A0A7G1KM99](https://www.uniprot.org/uniprotkb/A0A7G1KM99/entry) | 46.8 | 4.02e-42 | 91 |
| *Streptomyces finlayi* | [A0A7G7BW12](https://www.uniprot.org/uniprotkb/A0A7G7BW12/entry) | 44.1 | 4.11e-44 | 94 |
| *Streptomyces genisteinicus* | [A0A7H0HNM6](https://www.uniprot.org/uniprotkb/A0A7H0HNM6/entry) | 49.3 | 4.42e-48 | 95 |
| *Streptomyces xanthii* | [A0A7H1BFP7](https://www.uniprot.org/uniprotkb/A0A7H1BFP7/entry) | 46.2 | 4.25e-46 | 95 |
| *Streptomyces* sp. | [A0A7H8IHW3](https://www.uniprot.org/uniprotkb/A0A7H8IHW3/entry) | 43.9 | 4.22e-45 | 91 |
| *Mycolicibacterium duvalii* | [A0A7I7JU27](https://www.uniprot.org/uniprotkb/A0A7I7JU27/entry) | 76.4 | 8.14e-105 | 99 |
| *Mycolicibacterium arabiense* | [A0A7I7S6A3](https://www.uniprot.org/uniprotkb/A0A7I7S6A3/entry) | 71.5 | 7.82e-100 | 98 |
| *Mycobacterium botniense* | [A0A7I9XYW0](https://www.uniprot.org/uniprotkb/A0A7I9XYW0/entry) | 65.4 | 7.33e-93 | 97 |
| *Streptomyces fulvorobeus* | [A0A7J0C1G3](https://www.uniprot.org/uniprotkb/A0A7J0C1G3/entry) | 44.1 | 4.13e-44 | 91 |
| *Streptomyces smaragdinus* | [A0A7K0CPV8](https://www.uniprot.org/uniprotkb/A0A7K0CPV8/entry) | 44.1 | 4.11e-44 | 97 |
| *Actinomadura litoris* | [A0A7K1LCR6](https://www.uniprot.org/uniprotkb/A0A7K1LCR6/entry) | 43.6 | 4.04e-43 | 99 |
| *Streptomyces* sp. | [A0A7K2KZZ4](https://www.uniprot.org/uniprotkb/A0A7K2KZZ4/entry) | 45.9 | 4.02e-42 | 92 |
| *Streptomyces* sp. | [A0A7L4XYF5](https://www.uniprot.org/uniprotkb/A0A7L4XYF5/entry) | 46.4 | 6.20e-48 | 95 |
| *Streptomyces ferrugineus* | [A0A7M2T152](https://www.uniprot.org/uniprotkb/A0A7M2T152/entry) | 45.5 | 4.31e-47 | 100 |
| *Streptomyces* sp. | [A0A7M3LRJ1](https://www.uniprot.org/uniprotkb/A0A7M3LRJ1/entry) | 46.7 | 4.44e-49 | 94 |
| *Thermomonospora cellulosilytica* | [A0A7W3N0P9](https://www.uniprot.org/uniprotkb/A0A7W3N0P9/entry) | 45.4 | 4.11e-44 | 96 |
| *Streptomyces griseostramineus* | [A0A7W7M248](https://www.uniprot.org/uniprotkb/A0A7W7M248/entry) | 44.6 | 3.92e-41 | 92 |
| *Streptomyces olivoverticillatus* | [A0A7W7PJM2](https://www.uniprot.org/uniprotkb/A0A7W7PJM2/entry) | 46.4 | 4.33e-47 | 93 |
| *Lipingzhangella halophila* | [A0A7W7RMV1](https://www.uniprot.org/uniprotkb/A0A7W7RMV1/entry) | 46.9 | 4.27e-46 | 98 |
| *Streptomyces morookaense* | [A0A7Y7B221](https://www.uniprot.org/uniprotkb/A0A7Y7B221/entry) | 45 | 4.20e-45 | 93 |
| *Actinophytocola xinjiangensis* | [A0A7Z0WJN7](https://www.uniprot.org/uniprotkb/A0A7Z0WJN7/entry) | 44.9 | 3.91e-41 | 96 |
| *Planomonospora venezuelensis* | [A0A841DG50](https://www.uniprot.org/uniprotkb/A0A841DG50/entry) | 46.8 | 4.07e-43 | 94 |
| *Streptosporangium sandarakinum* | [A0A852UZM3](https://www.uniprot.org/uniprotkb/A0A852UZM3/entry) | 46.9 | 4.16e-45 | 99 |
| *Spirilliplanes yamanashiensis* | [A0A8J3YCH6](https://www.uniprot.org/uniprotkb/A0A8J3YCH6/entry) | 49.3 | 4.43e-49 | 98 |
| *Catenulispora acidiphila* | [C7QBD5](https://www.uniprot.org/uniprotkb/C7QBD5/entry) | 44.4 | 3.88e-40 | 93 |
| *Streptomyces venezuelae* | [F2RA35](https://www.uniprot.org/uniprotkb/F2RA35/entry) | 47.4 | 4.21e-45 | 94 |
| *Patulibacter medicamentivorans* | [H0E464](https://www.uniprot.org/uniprotkb/H0E464/entry) | 49.8 | 4.97e-57 | 99 |
| *Streptomyces davaonensis* | [K4R6D4](https://www.uniprot.org/uniprotkb/K4R6D4/entry) | 44 | 4.26e-46 | 91 |
| *Mycobacterium* sp. | [L0J1J6](https://www.uniprot.org/uniprotkb/L0J1J6/entry) | 72.7 | 7.81e-100 | 98 |
| *Mycolicibacterium cosmeticum* | [W9AVV1](https://www.uniprot.org/uniprotkb/W9AVV1/entry) | 67.8 | 7.40e-94 | 100 |
| *Myxococcales bacterium* | KPK14096.1 | 65.57 | 3.00e-79 | 86 |
| *Candidatus Dormibacteraeota bacterium* | MBV9100674.1 | 48.8 | 3.00e-52 | 98 |
| *Deltaproteobacteria bacterium* | TMB00698.1 | 47.47 | 8.00e-49 | 99 |
| *Acidobacteriota bacterium* | MBI4470031.1 | 47.57 | 6.00e-48 | 96 |
| *Deltaproteobacteria bacterium* | TMA50552.1 | 47.47 | 2.00e-47 | 99 |
| *Mesorhizobium* sp. | TPN29283.1 | 44.76 | 5.00e-44 | 98 |
| *Chloroflexota bacterium* | TMF15404.1 | 44.39 | 8.00e-41 | 96 |
| *Acidobacteriota bacterium* | MDE3069982.1 | 43.6 | 3.00e-39 | 99 |
| *Candidatus Dormibacteraeota bacterium* | MBJ7597971.1 | 47.09 | 1.00e-37 | 80 |
| *Chloroflexota bacterium* | MDE3094418.1 | 41.84 | 1.00e-33 | 90 |
| *Candidatus Dormibacteraeota bacterium* | MBO0684770.1 | 46.67 | 3.00e-33 | 83 |
| *Desulfotalea psychrophila* | WP_041277534.1 | 41.81 | 3.00e-32 | 81 |
| *Pseudomonadales bacterium* | MCG3169746.1 | 44.07 | 4.00e-32 | 81 |
| *Desulfotalea psychrophila* | CAG35225.1 | 41.81 | 6.00e-32 | 81 |
| *Reticulibacter mediterranei* | WP_220210729.1 | 43.01 | 1.00e-31 | 85 |
| *Polyangiaceae bacterium* | MBK8255068.1 | 43.09 | 2.00e-31 | 83 |
| *Betaproteobacteria bacterium* | OGA07993.1 | 42.63 | 2.00e-31 | 87 |
| *Dictyobacter kobayashii* | WP_126557608.1 | 41.12 | 3.00e-31 | 90 |
| *Microbacter* sp. | MVZ91645.1 | 43.81 | 4.00e-31 | 90 |
| *Acidobacteriota bacterium* | TDI31627.1 | 43.16 | 1.00e-30 | 87 |
| *Acidobacteriota bacterium* | MCZ6599416.1 | 42.23 | 1.00e-30 | 95 |
| *Candidatus Dormibacteraeota bacterium* | NNM96725.1 | 44.26 | 2.00e-30 | 86 |
| *Candidatus Dormibacteraeota bacterium* | MDA8393919.1 | 40.29 | 4.00e-30 | 96 |
| *Dictyobacter kobayashii* | GCE24393.1 | 43.18 | 8.00e-30 | 80 |
| *Mycolicibacterium chubuense* | [A0A0J6WM91](https://www.uniprot.org/taxonomy/1800) | 67 | 7.01e-88 | 90 |
| *Mycolicibacterium fortuitum* | [A0A0N9XRF5](https://www.uniprot.org/taxonomy/1766) | 61.2 | 6.47e-80 | 97 |
| *Mycolicibacterium wolinskyi* | A0A132PIS4 | 60.9 | 1.4e-80 | 98 |
| *Mycobacterium* sp. | A0A1A2G740 | 61.2 | 1.00e-80 | 96 |
| *Mycobacterium holsaticum* | A0A1E3RZN9 | 62.3 | 7.2e-86 | 99 |
| *Mycobacterium doricum* | A0A1X1T7X9 | 65.8 | 5.4e-86 | 98 |
| *Mycobacterium fragae* | A0A1X1UPL1 | 56.7 | 5.80e-70 | 96 |
| *Mycobacterium dioxanotrophicus* | A0A1Y0C7A1 | 61.2 | 2.8e-80 | 97 |
| *Nocardia mexicana* | A0A370H9K2 | 57.3 | 1.9e-71 | 98 |
| *Mycobacterium helveticum* | A0A557XZW2 | 55.7 | 2.5e-58 | 99 |
| *Mycolicibacterium phlei* | A0A5N5UXL6 | 57.9 | 6.02e-75 | 95 |
| *Mycolicibacterium vanbaalenii* | A0A5S9R517 | 66 | 1.6e-94 | 99 |
| *Mycolicibacterium poriferae* | A0A6N4VD83 | 94.1 | 7.9e-138 | 99 |
| *Trebonia kvetii* | A0A6P2BWD1 | 50.8 | 3.4e-60 | 94 |
| *Nocardia wallacei* | A0A7G1KM37 | 52.7 | 4.5e-65 | 99 |
| *Mycolicibacterium duvalii* | A0A7I7K174 | 61.9 | 1.6e-82 | 99 |
| *Nocardia macrotermitis* | A0A7K0D867 | 51.2 | 3.2e-65 | 99 |
| *Nocardia transvalensis* | A0A7W9PLK1 | 55.1 | 1.2e-66 | 98 |
| *Mycolicibacterium smegmatis* | A0QVN1 | 59 | 8.5e-78 | 97 |
| *Thermobispora bispora* | D6Y5L2 | 58.6 | 8.4e-80 | 99 |
| *Mycolicibacterium rhodesiae* | [G8RVS3](https://www.uniprot.org/taxonomy/710685) | 59.9 | 6.41e-79 | 97 |
| *Mycobacterium chubuense* | I4BHS8 | 65.8 | 6.8e-91 | 99 |
| *Nocardia brasiliensis* | [K0ER09](https://www.uniprot.org/taxonomy/1133849) | 53.8 | 5.31e-62 | 96 |
| *Mycolicibacterium hassiacum* | [K5B8H4](https://www.uniprot.org/taxonomy/1122247) | 59.9 | 6.21e-76 | 93 |
| *Mycolicibacterium poriferae* | ZYF656_4275U | 32.231 | 1.24e-09 | 54 |

**Table S7. Environmental metagenomes used in this study.**

| Metagenome | Genome ID | Gene count |
| --- | --- | --- |
| Seawaters (<200 m) | 3300021365 | 51,527,838 |
|  | 3300017799 |  |
|  | 3300020175 |  |
|  | 3300023086 |  |
|  | 3300021416 |  |
|  | 3300032691 |  |
|  | 3300037872 |  |
|  | 3300032559 |  |
|  | 3300032630 |  |
|  | 3300035205 |  |
|  | 3300024336 |  |
|  | 3300032480 |  |
|  | 3300037871 |  |
|  | 3300032673 |  |
| Seawaters (200-1000 m) | 3300040813 |  |
|  | 3300037866 |  |
|  | 3300028045 |  |
|  | 3300023276 |  |
|  | 3300027861 |  |
|  | 3300022916 |  |
|  | 3300037802 |  |
|  | 3300037870 |  |
|  | 3300037801 |  |
|  | 3300027996 |  |
|  | 3300040814 |  |
|  | 3300035163 |  |
|  | 3300035206 |  |
|  | 3300035100 |  |
|  | 3300023112 |  |
| Seawaters (>1000 m) | 3300035403 |  |
|  | 3300037621 |  |
|  | 3300035400 |  |
|  | 3300035404 |  |
|  | 3300034629 |  |
|  | 3300034654 |  |
|  | 3300037581 |  |
|  | 3300035399 |  |
|  | 3300037573 |  |
|  | 3300034655 |  |
|  | 3300037557 |  |
|  | 3300035402 |  |
|  | 3300034628 |  |
|  | 3300035401 |  |
|  | 3300035286 |  |
| Marine sediment | 3300009788 | 50,135,914 |
|  | 3300034302 |  |
|  | 3300037450 |  |
|  | 3300038548 |  |
|  | 3300037246 |  |
|  | 3300005590 |  |
|  | 3300027828 |  |
|  | 3300005920 |  |
|  | 3300009529 |  |
|  | 3300027967 |  |
|  | 3300024263 |  |
|  | 3300038629 |  |
|  | 3300034301 |  |
| Lake water | 3300045144 | 8,514,210 |
|  | 3300029268 |  |
|  | 3300021092 |  |
|  | 3300043465 |  |
|  | 3300045010 |  |
|  | 3300044987 |  |
|  | 3300045018 |  |
|  | 3300020220 |  |
|  | 3300043775 |  |
|  | 3300037829 |  |
|  | 3300031884 |  |
|  | 3300032881 |  |
|  | 3300045009 |  |
| Lake sediment | 3300035198 | 6,922,166 |
|  | 3300031999 |  |
|  | 3300016621 |  |
|  | 3300036761 |  |
|  | 3300031834 |  |
|  | 3300016581 |  |
|  | 3300016609 |  |
| Marsh sediment | 3300031643 | 2,753,065 |
|  | 3300031351 |  |
|  | 3300037399 |  |
|  | 3300031537 |  |
|  | 3300031585 |  |
|  | 3300031653 |  |
|  | 3300031276 |  |
|  | 3300031551 |  |
| Soils | 3300020795 | 6,333,561 |
|  | 3300020909 |  |
|  | 3300026481 |  |
|  | 3300043690 |  |
|  | 3300030606 |  |
|  | 3300034170 |  |
|  | 3300036859 |  |
|  | 3300036827 |  |
| Hydrothermal vent | 3300019457 | 383,001 |
|  | 3300019450 |  |
|  | 3300019455 |  |
|  | 3300019447 |  |
|  | 3300019452 |  |
|  | 3300019446 |  |
|  | 3300019453 |  |
|  | 3300019448 |  |
|  | 3300019443 |  |
|  | 3300019439 |  |
|  | 3300019440 |  |
|  | 3300019456 |  |
|  | 3300019454 |  |
|  | 3300019451 |  |
|  | 3300019395 |  |
| Cold spring | SRR12623522 (S7) | 11,423,434 |
|  | SRR12623521 (S6) |  |
|  | SRR12623520 (S5) |  |
|  | SRR12623519 (S4) |  |

**Reference**

1. C. Yanisch-Perron, J. Vieira, and J. Messing, Improved M13 phage cloning vectors and host strains: nucleotide sequences of the M13mp18 and pUC19 vectors. *Gene*, 1985. 33(1): 103-19.

2. D. J. MacNeil, J. L. Occi, K. M. Gewain, et al., Complex organization of the Streptomyces avermitilis genes encoding the avermectin polyketide synthase*.* *Gene*, 1992. 115(1-2): 119-25.

3. R. E. Cobb, Y. Wang, and H. Zhao, High-efficiency multiplex genome editing of Streptomyces species using an engineered CRISPR/Cas system. *ACS Synthetic Biology*, 2015. 4(6): 723-8.

4. S. Sioud, B. Aigle, I. Karray-Rebai, et al., Integrative Gene Cloning and Expression System for Streptomyces sp. US 24 and Streptomyces sp. TN 58 Bioactive Molecule Producing Strains. *BioMed Research International*, 2009. 2009(1): 464986.

5. H.-J. Hong, M. I. Hutchings, L. M. Hill, M. J. Buttner, The Role of the Novel Fem Protein VanK in Vancomycin Resistance in Streptomyces coelicolor. *Journal of Biological Chemistry*, 2005. 280(13): 13055-13061.
